# Process-Induced Metabolite Remodeling of *Tripterygium* Glycosides and Its Association with Circulating Prototype Constituents

**DOI:** 10.3390/metabo16070476

**Published:** 2026-07-07

**Authors:** Tao Zhang, Junchao Liu, Huiyi Wen, Jianqun Liu

**Affiliations:** Key Laboratory of Modern Preparation of TCM, Ministry of Education, Jiangxi University of Chinese Medicine, Nanchang 330004, China; zhangtao81@jxutcm.edu.cn (T.Z.); liujunchao@jxutcm.edu.cn (J.L.); wenhuiyi@jxutcm.edu.cn (H.W.)

**Keywords:** *Tripterygium* glycosides, metabolite profiling, circulating prototype constituents, single-time-point serum profiling, process-responsive quality markers, roasting, dealkalization, UPLC-Q-TOF-MS/MS

## Abstract

**Background/Objectives**: *Tripterygium* glycosides (TG) are used to treat inflammatory and autoimmune diseases, but their clinical application is limited by toxicity and the lack of process-responsive quality markers. This study examined whether roasting and dealkalization remodel the TG metabolite profile and alter the post-dose serum profile of circulating prototype constituents. **Methods**: Self-prepared TG, roasted TG (RTG), roasted–dealkalized TG (RDTG), and five marketed products were profiled by ultra-performance liquid chromatography coupled with quadrupole time-of-flight tandem mass spectrometry (UPLC-Q-TOF-MS/MS). Seven representative compounds were quantified by validated high-performance liquid chromatography (HPLC). Rat serum after oral administration was analyzed to compare circulating prototype constituents. **Results**: We characterized 243 constituents in material samples and 63 circulating prototype constituents in serum. Roasting primarily reshapes the profiles of diterpenoids and triterpenoids. Celastrol was not detected in the RTG and RDTG material samples, nor in the corresponding single-time-point serum profiles under the current analytical conditions. In contrast, wilforlide A exhibited an increase in material samples. Dealkalization preferentially reduced alkaloid-related constituents, including wilforine in material samples and tripterygiumine T in serum. **Conclusions**: Integrated material profiling, targeted quantification, and serum prototype analysis identified candidate process-responsive markers for processed TG preparations. Because the serum study was based on relative signal intensities rather than full pharmacokinetics, these markers require further pharmacokinetic and toxicological validation.

## 1. Introduction

*Tripterygium wilfordii* Hook. f. (*T. wilfordii*), a member of the Celastraceae family, has a long history of use in traditional Chinese medicine, and its preparations are currently used for inflammatory and autoimmune diseases, particularly rheumatoid arthritis [1]. Its primary pharmaceutical form, *Tripterygium* glycoside (TG), is a multicomponent extract particularly effective for rheumatoid arthritis due to its anti-inflammatory and immunoregulatory properties. However, TG’s clinical application is limited by its narrow therapeutic window and known toxicities affecting the liver, kidneys, and reproductive system, among others [2,3]. This risk–benefit profile is intricately linked to its complex chemistry. While diterpenoids, triterpenoids, and sesquiterpene pyridine alkaloids contribute to its pharmacological effects, they are also associated with adverse reactions [4,5,6]. The chemical complexity complicates quality evaluation. Research on marketed *Tripterygium* products has revealed significant batch-to-batch and manufacturer-dependent variations, indicating that product quality cannot be adequately assessed using just one or two marker compounds [7,8]. A more effective approach involves characterizing the global chemical profile, identifying constituents responsive to processing, and verifying representative markers through targeted quantification. Similar integrated profiling and quantification strategies have proven beneficial for the holistic quality evaluation of other herbal medicines [9,10].

Pharmaceutical processing is commonly employed in traditional medicine to minimize toxicity while maintaining efficacy [11]. For botanical matrices abundant in alkaloids and terpenoids, controlled heating can modify chemical profiles through processes such as hydrolysis, oxidation, decomposition, and condensation [12,13]. In the case of *T. wilfordii*, roasting the root xylem at 200 °C significantly altered the extract’s composition, especially affecting alkaloids and triterpenoids. Celastrol, a quinone methide triterpenoid associated with toxicity, has been reported to exhibit a high sensitivity to roasting. It undergoes thermal transformation, which includes partial conversion to 1-hydroxy-2,5,8-trimethyl-9-fluorenone [14,15]. These transformations, along with reductions in total alkaloids, contributed to the depletion of specific toxicologically relevant chemical markers following the roasting process. However, sesquiterpene pyridine alkaloids remain a significant safety concern due to their association with hepatorenal toxicity. Therefore, a subsequent dealkalization step, utilizing acid–base dissociation, may offer a targeted approach to remove alkaloid-rich fractions. Sequential roasting and dealkalization are anticipated to complementarily remodel the TG matrix, but this hypothesis requires systematic chemical and serum-level validation.

The rationale for combining roasting and dealkalization is clear, yet the molecular changes these processes induce have not been fully mapped. It remains unclear which constituents are altered, enriched, or removed during thermal roasting and which are further affected by acidic dealkalization. Additionally, TG products from various manufacturers exhibit significant differences in chemical profiles and biological effects [6,7]. Therefore, including representative marketed products as external comparators can help contextualize the chemical features of self-prepared TG preparations and evaluate whether candidate quality markers are informative across different commercial product profiles. Another unresolved issue is the connection between the in vitro chemical profile and systemic exposure. A constituent abundant in the extract may not necessarily overcome gastrointestinal absorption, first-pass metabolism, and efflux barriers to become a circulating prototype. Therefore, an analytical strategy is needed to link process-induced changes in the material basis with changes in circulating prototype constituents.

We hypothesized that roasting would primarily alter diterpenoid- and triterpenoid-related metabolites, while acidic dealkalization would selectively reduce sesquiterpene pyridine alkaloids. We also anticipated that these process-induced changes would be evident in circulating prototype exposure following oral administration. To test these hypotheses, this study employed a three-layer analytical approach centered on metabolites. First, we comprehensively profiled self-prepared TG, roasted TG (RTG), roasted–dealkalized TG (RDTG), and five marketed products using ultra-performance liquid chromatography coupled with quadrupole time-of-flight tandem mass spectrometry (UPLC-Q-TOF-MS/MS). Second, we quantified seven representative markers using high-performance liquid chromatography (HPLC) to validate key changes responsive to the processes. Third, we characterized prototype metabolites in rat serum post-oral administration to assess whether changes at the material level were reflected in systemic exposure. By integrating material profiling, targeted quantification, and serum prototype analysis, this study proposes a framework for evaluating processed TG preparations and nominates candidate process-responsive markers for further validation.

## 2. Materials and Methods

### 2.1. Chemicals and Reagents

Roots of *T. wilfordii* were collected from 5- to 8-year-old plants in Xiangdong District, Pingxiang City, Jiangxi Province, China, during July–August 2024. The plant material was authenticated by Professor Xiaomei Fu from Jiangxi University of Chinese Medicine. Five commercial *Tripterygium* glycoside tablet products, labeled MTG1-MTG5, were obtained from various pharmaceutical companies: Zhejiang Deende Pharmaceutical Co., Ltd. (Shaoxing, China; batch 2508107B); Yuanda Pharmaceutical Huangshi Feiyun Co., Ltd. (Huangshi, China; batch 20250502); Shanghai Fudan Forward Pharmaceutical Co., Ltd. (Shanghai, China; batch 240705); Jiangsu Meitong Pharmaceutical Co., Ltd. (Taizhou, China; batch 250705); and Hunan Qianjin Xieli Pharmaceutical Co., Ltd. (Zhuzhou, China; batch 241101). These marketed products were included as external commercial comparators to evaluate inter-product chemical variability and to assess the applicability of the proposed multi-marker quality-evaluation strategy.

Reference standards used for structural annotation and quantification had purities of at least 98% by peak-area normalization. Triptolide, celastrol, tripdiolide, triptonide, triptophenolide, demethylzeylasteral, 1-hydroxy-2,5,8-trimethyl-9-fluorenone, wilforine, wilfordine, wilforgine, celacarfurine, celabenzine, celallocinnine, and tripfordine A were isolated and purified in our laboratory by chromatographic methods adapted, with minor optimization, from previously reported phytochemical studies of *T. wilfordii* [16,17,18]. Wilforlide A (batch 111597-202006, purity > 98%) was sourced from the National Institutes for Food and Drug Control of China (Beijing, China). We obtained analytical-grade ethyl acetate and methanol from Xilong Scientific Co., Ltd. (Shantou, China). Mass spectrometry-grade methanol, acetonitrile, and formic acid were purchased from Merck (Darmstadt, Germany) and Maclin Biochemical Technology Co., Ltd. (Shanghai, China). Ultrapure water was prepared using a Milli-Q system (Merck Millipore, Burlington, MA, USA), and carboxymethylcellulose sodium (CMC-Na) was supplied by Shanghai Shanpu Chemical Co., Ltd. (Shanghai, China).

### 2.2. Preparation of TG, RTG, and RDTG

TG was prepared by an ethanol extraction–ethyl acetate partitioning procedure adapted with minor modifications from previously reported methods for *T. wilfordii* or TG and from our prior processing study [5,14,15]. Briefly, crude root powder was reflux extracted three times with 70% ethanol using a solvent volume five times that of the powder. The three extraction steps lasted 1.5, 1.5, and 1 h, respectively. The combined extracts were concentrated and partitioned three times with 1.5 volumes of ethyl acetate to enrich the medium-polarity terpenoid- and alkaloid-containing fraction. The ethyl acetate fraction was evaporated to dryness, redissolved in methanol, and further purified by methanol–water gradient elution. The 80% and 100% methanol–water eluates, which contained the major lipophilic TG-related constituents, were collected, concentrated, and freeze-dried to yield TG.

RTG was prepared from roasted roots following our previous processing study [14]. In summary, woody root xylem sections were size-fractionated, wrapped in tinfoil, and covered with yellow clay. The samples were air-dried in a shaded, ventilated area before being roasted in an oven at 200 °C for 45 min. Once cooled, the clay and foil were removed, and the roasted material was powdered and processed similarly to TG.

RDTG was prepared using the RTG extraction system by concentrating the alcohol extract and then partitioning it. The concentrated organic extract was suspended and stirred with 3% dilute sulfuric acid. After phase separation, the acidic aqueous layer, which was expected to be enriched in protonated basic constituents, including sesquiterpene pyridine alkaloid-related compounds, was discarded. This acid-washing step was repeated three times to further deplete the alkaloids. The remaining ethyl acetate layer, which was relatively enriched in terpenoid-related constituents according to HPLC analysis of representative marker compounds, was washed with distilled water until the pH reached approximately 7.0. It was then processed as previously described to yield RDTG.

### 2.3. UPLC-Q-TOF-MS/MS Analysis of Material Samples

#### 2.3.1. Preparation of Material Samples

For high-resolution material profiling, 100 mg of TG, RTG, or RDTG powder was dissolved in 2 mL of mass spectrometry-grade methanol. The solution was then ultrasonicated and filtered through a 0.22 μm microporous membrane. To prepare commercial tablets, 10 tablets per batch were ground, each containing approximately 10 mg of total glycosides. The resulting powder was extracted with 20 mL of ethyl acetate under ultrasonication for 45 min. After evaporation to dryness, the extract was reconstituted in 2 mL of methanol.

#### 2.3.2. UPLC-Q-TOF-MS/MS Conditions

Chromatographic separation was conducted using a Shimadzu Nexera X2 LC-30A system (Shimadzu, Kyoto, Japan) paired with an AB SCIEX TripleTOF 5600+ high-resolution mass spectrometer (SCIEX, Framingham, MA, USA). A Waters ACQUITY BEH C18 column (2.1 mm × 50 mm, 1.7 μm; Waters, Milford, MA, USA) was maintained at 40 °C. The mobile phases consisted of 0.1% formic acid in water (A) and acetonitrile (B). The gradient program was as follows: from 0.01 to 20 min, 10–32% B; from 20 to 45 min, 32–90% B; from 45 to 47 min, 90% B; from 47 to 47.1 min, 90–10% B; and from 47.1 to 50 min, 10% B.

Mass spectrometric detection employed an electrospray ionization source in positive ion mode, scanning a mass range of *m*/*z* 50–1500. The source temperature was set to 500 °C, with an ion spray voltage of 5500 V. Collision energy was maintained at 35 eV, with a spread of 15 eV. Nebulizer and auxiliary gases were both set at 50 kPa, while the curtain gas was at 40 kPa, using nitrogen as the gas source.

### 2.4. Compound Annotation Strategy and Chemometric Analysis

A comprehensive database of TG-related compounds has been compiled using CNKI, SciFinder, PubMed, TCMSP, and published literature. Compound annotation confidence is assigned based on the level of supporting evidence. Compounds are classified as standard-confirmed when their retention time, accurate mass, and MS/MS fragmentation behavior match those of authentic reference standards analyzed under identical analytical conditions. Compounds lacking authentic-standard confirmation are classified as tentatively annotated when their assignments are supported by accurate mass, characteristic MS/MS fragment ions, retention behavior, database information, and literature data. Consequently, the terms “confirmed” or “identified by authentic standards” are applied exclusively to standard-matched compounds, while “tentatively annotated” refers to MS-based assignments without authentic standards.

MarkerView 1.2.1.1 facilitates peak extraction, peak alignment, and statistical feature extraction. The exported feature matrix includes retention time, *m*/*z* value, and peak area. Prior to statistical analysis, isotopic ions, duplicate ions derived from adducts, and redundant signals originating from the same chromatographic peak were eliminated. For chemometric analysis, peak areas were normalized to mitigate differences in total signal intensity among samples. Missing values were addressed using a small-value imputation strategy when the feature was detected in the majority of samples within at least one group; features with excessive missing values or unstable detection were excluded. The resulting data matrix underwent log transformation to reduce heteroscedasticity and was subsequently scaled before multivariate analysis. Principal Component Analysis (PCA) was conducted as an unsupervised method to assess sample clustering and the overall data structure. Partial Least Squares Discriminant Analysis (PLS-DA) was then employed to visualize group discrimination and identify variables contributing to separation. The robustness of the PLS-DA model was assessed using 200-permutation testing. Candidate differential constituents were identified using VIP ≥ 1 from the PLS-DA model in conjunction with nominal *p* ≤ 0.05 from univariate statistical analysis.

### 2.5. HPLC Method Development and Validation for Representative Markers

#### 2.5.1. Preparation of Sample and Reference Solutions for HPLC Analysis

The seven-marker module included tripdiolide, triptolide, triptophenolide, demethylzeylasteral, wilforine, celastrol, and wilforlide A.

Sample solutions for HPLC were prepared as described above for TG, RTG, RDTG, and the five commercial tablets. The mixed reference solution contained wilforlide A (85 μg/mL), triptolide (153 μg/mL), celastrol (146 μg/mL), tripdiolide (72 μg/mL), triptophenolide (137 μg/mL), demethylzeylasteral (53 μg/mL), and wilforine (102 μg/mL).

#### 2.5.2. HPLC Conditions

HPLC analysis was conducted using an Agilent 1260 system (Agilent Technologies, Santa Clara, CA, USA) equipped with a Shim-pack C18 column (4.6 mm × 250 mm, 5 μm; Shimadzu, Kyoto, Japan). The mobile phases consisted of acetonitrile (A) and 0.1% formic acid in water (B). The gradient was as follows: 0–15 min, 20–40% A; 15–18 min, 40% A; 18–52 min, 40–80% A; 52–56 min, 80% A; 56–64 min, 80–100% A; and 64–66 min, 100–20% A. The flow rate was maintained at 1.0 mL/min. Variable-wavelength detection was set at 218 nm for 0–20 min, 270 nm for 20–37 min, 230 nm for 37–38 min, 400 nm for 38–55 min, and 210 nm for 55–66 min. The column temperature was held at 30 °C, and the injection volume was 10 μL.

The method validation encompassed assessments of linearity, precision, repeatability, stability, and recovery. Experimental data are reported as mean ± standard deviation (mean ± SD). Statistical analyses were performed using SPSS V32 and GraphPad Prism 10.0. For two-group comparisons, we employed Student’s *t*-test, while one-way ANOVA with Dunnett’s T3 post hoc test was utilized for multi-group comparisons. All tests were two-sided, and *p* < 0.05 was considered statistically significant. Exact *p*-values are reported where possible.

### 2.6. Animal Administration and Serum Sample Preparation

SPF-grade male Sprague Dawley rats, weighing 180–200 g, were obtained from Hunan SJA Laboratory Animal Co., Ltd. (Changsha, China; license no. SCXK [Xiang] 2021-0002). The animals underwent a 7-day acclimation period under controlled environmental conditions, maintaining a temperature of 22–28 °C and a relative humidity of 50–70%. All animal procedures received approval from the institutional animal ethics committee (approval no. HLK-20250902-005).

Following acclimation, the rats were randomly assigned to five groups, each consisting of six rats: the control group, TG group, RTG group, RDTG group, and MTG-1 group. MTG-1 served as a representative marketed TG preparation and acted as the commercial product control. TG, RTG, RDTG, and MTG-1 were suspended in 5% CMC-Na at a concentration of 9.45 mg/mL. The four preparations were administered orally to the rats once daily for seven consecutive days at a dose of 94.5 mg/kg, which corresponds to ten times the rat-equivalent clinical dose. Rats in the control group received an equal volume of 5% CMC-Na.

Prior to the final administration, the animals were fasted for 12 h while having free access to water. Blood samples were collected one hour after the final oral administration. The rats were deeply anesthetized via intraperitoneal injection of 1.5% sodium pentobarbital before blood collection and were subsequently euthanized in accordance with the approved animal protocol. Serum was separated by centrifugation and stored at −80 °C until UPLC-Q-TOF-MS/MS analysis.

For each individual serum sample, an 80 μL aliquot was transferred into a 2 mL tube for protein precipitation. Methanol–acetonitrile (3:1) was added in three volumes to the mixture, which was then vortexed for 3 min and centrifuged at 14,000 r/min for 15 min. The supernatant was transferred to a new tube and dried under nitrogen. The residue was reconstituted in 100 μL of methanol, vortexed for another 3 min, and centrifuged again at 14,000 r/min for 15 min at 4 °C. The resulting supernatant was injected for analysis. Pooled quality-control (QC) samples were prepared by taking 20 μL from each serum sample and processing them using the same method.

### 2.7. UPLC-Q-TOF-MS/MS Analysis of Serum Samples

Serum samples were separated on a Waters ACQUITY BEH C_18_ column (2.1 mm × 50 mm, 1.7 μm) at 40 °C with a flow rate of 0.25 mL/min and an injection volume of 2 μL. The mobile phases were 0.1% formic acid in water (A) and acetonitrile (B). The gradient was 0.01–8 min, 5–30% B; 8–12 min, 30–65% B; 12–20 min, 65–95% B; 20–22 min, 95% B; 22–22.1 min, 95–5% B; and 22.1–25 min, 5% B.

Mass spectrometric data were acquired in positive electrospray ionization mode over *m*/*z* 50–1250. The ion source temperature was 500 °C, the ion spray voltage was 5500 V, the collision energy was 35 eV, the collision energy spread was 15 eV, and the gas settings were the same as those used for the material-sample method.

### 2.8. Annotation and Chemometric Analysis of Circulating Prototype Constituents

Circulating prototype constituents are annotated using the same overall strategy applied to material samples. The serum analysis serves as a screening approach to compare prototype constituents detectable in serum at a fixed sampling time, rather than functioning as a pharmacokinetic study. Consequently, serum results are reported as relative UPLC-Q-TOF-MS/MS signal intensities measured 1 h after the final oral administration. Serum constituents that directly match available authentic standards are classified as standard-confirmed. In contrast, constituents identified by accurate mass, MS/MS fragments, retention behavior, and literature evidence are categorized as tentatively annotated.

PCA assesses clustering and the stability of quality-control samples, while PLS-DA visualizes group discrimination. Model robustness is evaluated through 200-permutation testing. Candidate differential serum-detectable prototype constituents are screened using VIP ≥ 1 and nominal *p* ≤ 0.05. Since the serum analysis represents an exploratory, single-time-point, relative signal-based screening analysis and no formal multiple-testing correction is applied, the differential serum features should be interpreted as candidate serum linkage signals that require further confirmation through targeted quantitative and pharmacokinetic studies.

## 3. Results

### 3.1. Material Metabolite Profiling of TG, RTG, and RDTG

**Figure 1:**
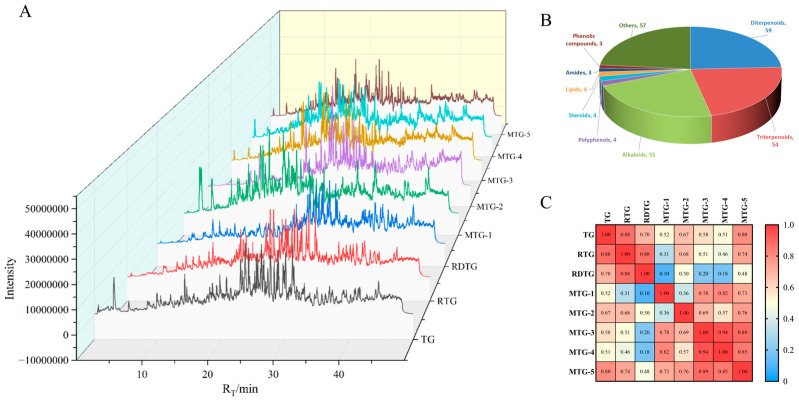
UPLC-Q-TOF-MS/MS-based material profiling of TG-related samples: (**A**) Representative base peak chromatograms of TG, RTG, RDTG, and five marketed TG products. (**B**) Chemical-class distribution of annotated constituents. The numbers indicate the number of compounds. (**C**) Spearman correlation matrix of the eight TG-related material samples. The color scale in panel C represents the Spearman correlation coefficient. TG, self-prepared *Tripterygium* glycosides; RTG, roasted TG; RDTG, roasted–dealkalized TG; MTG1–MTG5, marketed *Tripterygium* glycoside products.

The in vitro UPLC-Q-TOF-MS/MS analysis identified the extensive chemical composition of TG and its process-derived products (Figure 1A). A total of 243 compounds were characterized across TG, RTG, RDTG, and five commercial tablet products. Among them, 13 constituents were confirmed by comparison with authentic standards, whereas the remaining 230 constituents were tentatively annotated based on accurate mass, MS/MS fragmentation behavior, retention behavior, and literature data. These constituents included 59 diterpenoids, 54 triterpenoids, 55 alkaloids, 4 polyphenols, 4 lipids, 4 steroids, 3 phenolic compounds, 3 amides, and 57 other constituents (Figure 1B and Table S1). The findings confirm that TG is a chemically diverse preparation, primarily composed of diterpenoids, triterpenoids, and alkaloids, rather than just a simple glycoside system. Among the eight material samples, 124 constituents were consistently detected, suggesting a stable shared structural core despite noticeable inter-sample variations. This broad chemical coverage underscores the importance of adopting a holistic analytical strategy rather than relying solely on a minimal marker-based approach.

Detailed heatmaps of diterpenoids, triterpenoids, alkaloids, and other constituents were generated to compare chemical-class-specific variation among the TG-related samples and are provided in Supplementary Figures S1–S4. A Spearman correlation analysis was conducted on the eight TG-related material samples and is shown in Figure 1C. The three self-prepared samples (TG, RTG, and RDTG) demonstrated strong consistency in their chemical fingerprints, with correlation coefficients (ρ) of 0.88 for TG versus RTG and 0.88 for RTG versus RDTG. These findings suggest that roasting and dealkalization preserved the overall spectral framework while systematically altering the relative abundance of key constituents. In contrast, the five marketed samples exhibited greater variability. Among them, MTG3 and MTG4 were most similar to each other (ρ = 0.94), yet their chemical phenotypes differed from the self-prepared samples in the diterpenoid, triterpenoid, and alkaloid modules. This suggests potential differences in raw material batches, preparation processes, or quality standards among the product systems. This deep phenotypic divergence serves two purposes in our study design. First, it confirms pronounced manufacturer-dependent and batch-related compositional variability. Second, it provides a real-world baseline showing that a single-marker standard cannot accommodate the chemical heterogeneity of these products and thus directly supports the development of our multi-component, process-responsive marker strategy.

### 3.2. Process-Responsive Metabolite Remodeling Associated with Roasting and Dealkalization

**Figure 2:**
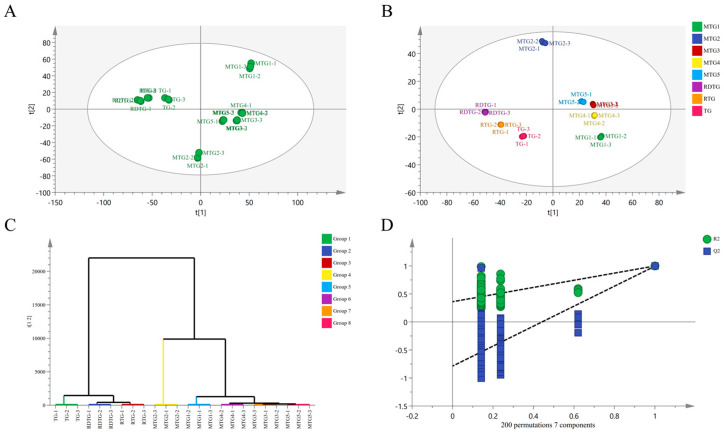
Chemometric analysis of material samples: (**A**) Principal component analysis (PCA) score plot. (**B**) Partial least-squares discriminant analysis (PLS-DA) score plot. (**C**) Hierarchical clustering analysis of TG-related samples. (**D**) The 200-permutation test of the PLS-DA model. In panel C, solid black lines indicate the original model values. In panel D, dashed lines indicate regression lines from permutation testing.

PCA demonstrated a clear distinction between the three self-prepared samples and the five marketed products (Figure 2A). The self-prepared samples—TG, RTG, and RDTG—clustered together, while the marketed products formed a separate group, indicating a difference in material composition. Within the self-prepared samples, RTG and RDTG were more closely related to each other than to TG, aligning with their sequential roasting and dealkalization processes. PLS-DA reinforced this pattern (Figure 2B), showing strong explanatory and predictive parameters (*R*^2^*Y* = 0.92, *Q*^2^ = 0.983). Among the commercial products, MTG-2 was most clearly separated from the remaining products, suggesting pronounced manufacturer- or batch-related divergence. Cluster analysis (Figure 2C) consistently grouped the samples in line with the PCA and PLS-DA findings. The 200-permutation test (Figure 2D) confirmed the model’s stability, as the permuted *R*^2^ and *Q*^2^ values were lower than the original values.

Differential screening, using VIP ≥ 1 and *p* ≤ 0.05, identified 56 distinct constituents between TG and RTG and 37 between RTG and RDTG (Table S2). A detailed heatmap of these candidate differential constituents is provided in Supplementary Figure S5.

#### 3.2.1. Roasting-Associated Changes

In the comparison between TG and RTG, the differential set primarily included 17 diterpenoids, 15 triterpenoids, and 8 alkaloids. This indicates that the most significant changes occurred in the diterpenoid and triterpenoid classes. Among the 59 annotated diterpenoid constituents in the material samples, 25 showed a greater than two-fold decrease in mean normalized UPLC-Q-TOF-MS/MS response intensity after roasting, defined as an RTG/TG response ratio of <0.50. Epoxidized diterpene lactones, such as tripdiolide and triptonide, decreased significantly, while some hydroxylated derivatives, like 5α-hydroxytriptonide, increased. This pattern suggests oxidative hydration, partial thermal degradation, and a redistribution of the diterpenoid profile during roasting. Polyphenolic constituents, including epigallocatechin, procyanidin B3, and catechin, also sharply decreased after roasting, aligning with the susceptibility of thermolabile and readily oxidizable polyphenols to high-temperature processing [19,20]. Notably, celastrol, a reactive quinone methide triterpenoid linked to toxicological concerns, was detected in TG but not in RTG or RDTG under the current UPLC-Q-TOF-MS/MS conditions. This finding aligns with previous reports indicating that celastrol is highly sensitive to roasting and can undergo thermal transformation, including partial conversion to 1-hydroxy-2,5,8-trimethyl-9-fluorenone [14,15]. The overall alkaloid profile showed only moderate suppression during roasting; among the 55 annotated alkaloid-related constituents in the material samples, 8 showed a greater than two-fold decrease in mean normalized UPLC-Q-TOF-MS/MS response intensity after roasting, defined as an RTG/TG response ratio of <0.50. Therefore, roasting induced selective degradation, conversion, and redistribution rather than a broad destruction of the TG chemical profile.

#### 3.2.2. Dealkalization-Associated Changes

In contrast, the comparison between RTG and RDTG revealed a more directional signature. Alkaloids constituted nearly half of the differential set, with 18 alkaloid components significantly reduced in RDTG, indicating dealkalization as a targeted removal step. Under acidic conditions, basic nitrogen-containing constituents can be protonated to form more water-soluble ionic species, thereby promoting their preferential partitioning into the acidic aqueous phase, which is subsequently discarded [12,16,21]. However, this partitioning process is not necessarily exclusive to sesquiterpene pyridine alkaloids, because other polar or ionizable constituents may also enter the acidic aqueous phase. Highly toxic alkaloids such as tripfordine A, celabenzine, wilfornine E, and chiapenine ES-IV were reduced from substantial peaks to trace or undetectable levels [17,22]. These findings suggest that acidic dealkalization selectively removed alkaloid-rich constituents while preserving much of the neutral terpenoid fraction in the organic phase. Nevertheless, because the discarded acidic aqueous phase was not comprehensively analyzed, the possible co-partitioning of other polar or ionizable constituents cannot be excluded.

### 3.3. Targeted HPLC Quantification of Process-Responsive Metabolite Markers

**Figure 3:**
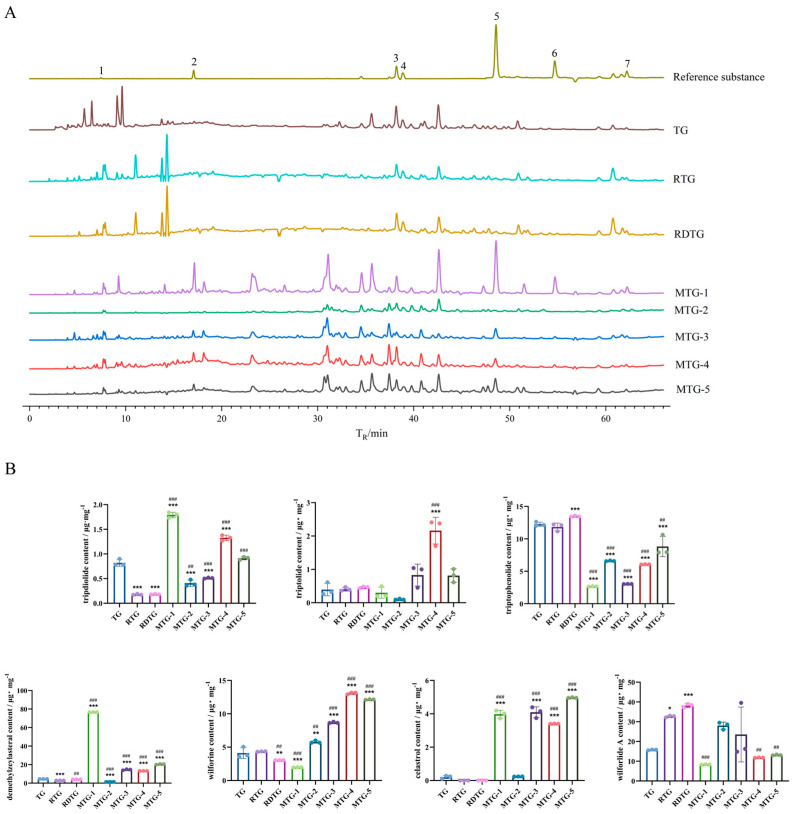
HPLC quantification of seven representative marker constituents in TG-related samples: (**A**) Representative HPLC chromatograms of mixed reference standards and sample extracts. (**B**) Contents of tripdiolide, triptolide, triptophenolide, demethylzeylasteral, wilforine, celastrol, and wilforlide A in TG, RTG, RDTG, and five marketed TG products. Data are presented as mean ± SD, *n* = 3. Compared with TG, * *p* < 0.05, ** *p* < 0.01, *** *p* < 0.001; compared with RTG, ^##^ *p* < 0.01, ^###^ *p* < 0.001. HPLC, high-performance liquid chromatography; TG, self-prepared *Tripterygium* glycosides; RTG, roasted TG; RDTG, roasted–dealkalized TG; MTG1–MTG5, marketed *Tripterygium* glycoside products; SD, standard deviation.

To convert relative mass spectrometric intensity shifts into quantitative data for quality control, seven representative markers were analyzed using HPLC-UV. The chromatograms indicated that the retention times of these compounds in various *Tripterygium* glycoside samples matched those of the reference standards, exhibiting satisfactory peak shapes without significant tailing (Figure 3A). All calibration curves demonstrated excellent linearity (r > 0.999), with the calibration equations and linear ranges detailed in Table 1. Method validation confirmed acceptable instrument precision, with relative standard deviations (RSDs) of 0.014–0.122% for relative retention time and 0.67–2.34% for peak area. Repeatability RSDs were between 1.15% and 2.78%; 24 h sample stability RSDs ranged from 2.52% to 2.97%; and recoveries were between 96.43% and 106.24%, with RSDs of 0.87–3.06%.

Table 2 and Figure 3B present the quantitative results for the seven indicator constituents. In TG, celastrol was quantified at 0.21 ± 0.09 μg/mg. However, after roasting at 200 °C for 45 min, celastrol fell below the detection limit in both RTG and RDTG. This finding is consistent with previous studies showing that celastrol is thermally responsive during roasting and can be partially converted to 1-hydroxy-2,5,8-trimethyl-9-fluorenone [14]. This conversion may mitigate risks linked to its electrophilic quinone methide pharmacophore [23]. Conversely, wilforlide A increased from 15.78 ± 0.20 μg/mg in TG to 32.68 ± 0.45 μg/mg in RTG and 38.17 ± 0.94 μg/mg in RDTG. Since wilforlide A remains stable below 200 °C, this rise likely results from enhanced extractability due to thermal disruption of the lignocellulosic matrix or release from bound or entrapped forms [18,24,25,26]. Further structural studies are necessary to elucidate potential precursor–product relationships. Triptolide showed relative stability, changing slightly from 0.40 ± 0.19 μg/mg in TG to 0.41 ± 0.06 μg/mg in RTG. This suggests that the roasting protocol effectively reduced certain toxic markers while preserving some bioactive diterpenoids.

HPLC data confirmed the impact of dealkalization. The sesquiterpene alkaloid wilforine decreased from 4.35 ± 0.03 μg/mg in RTG to 3.04 ± 0.01 μg/mg in RDTG, aligning with the alkaloid reduction observed in the mass spectrometric analysis. Additionally, tripdiolide levels dropped from 0.82 μg/mg in TG to 0.17 μg/mg in RTG. Demethylzeylasteral decreased from 4.60 μg/mg in TG to 3.02 μg/mg in RTG before rising to 4.11 μg/mg in RDTG.

The parallel quantification of five marketed preparations further supports their use as external comparators for evaluating commercial-product heterogeneity. The seven HPLC markers varied substantially among MTG1–MTG5. For example, demethylzeylasteral reached 76.71 ± 0.14 μg/mg in MTG1, which was more than sixteen times higher than in self-prepared TG, whereas MTG2 contained only 1.35 ± 0.03 μg/mg. Celastrol, wilforine, and wilforlide A also showed marked inter-product variation. These discrepancies likely reflect differences in raw-material selection, tissue sources, extraction and purification procedures, formulation methods, or manufacturer-specific quality-control strategies. Therefore, comparison with marketed products supports the need for a multi-marker quality-control strategy rather than reliance on a single marker.

### 3.4. Circulating Prototype Metabolites After Oral Administration

**Figure 4:**
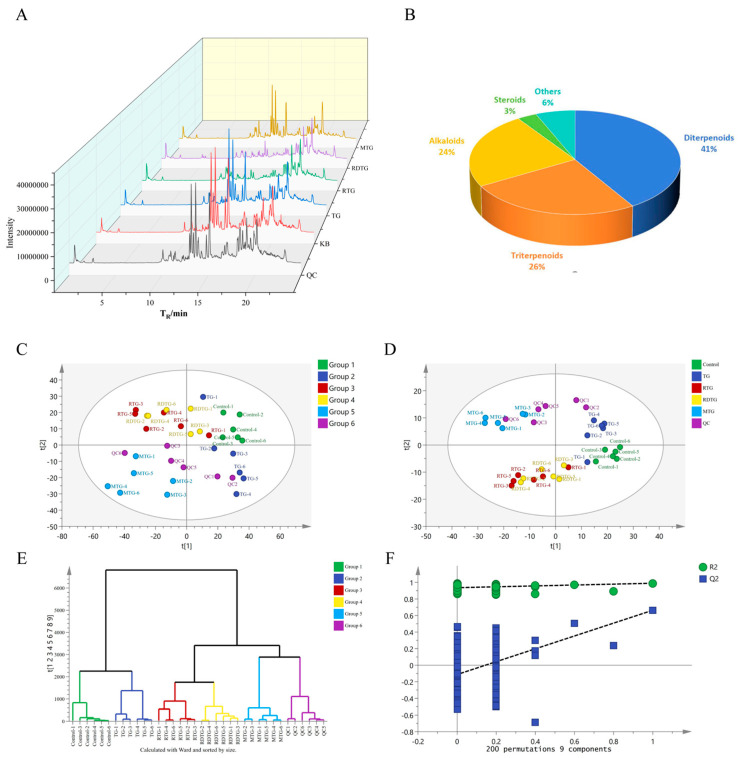
Single-time-point serum prototype screening after oral administration of TG-related preparations: (**A**) Representative serum base-peak chromatograms. (**B**) Chemical-class distribution of annotated serum-detectable prototype constituents. (**C**) Principal component analysis (PCA) score plot. (**D**) Partial least-squares discriminant analysis (PLS-DA) score plot. (**E**) Hierarchical clustering analysis of serum samples. (**F**) The 200-permutation test of the PLS-DA model. The serum results represent relative UPLC-Q-TOF-MS/MS signal intensities at a single sampling time point and should not be interpreted as pharmacokinetic parameters. TG, self-prepared *Tripterygium* glycosides; RTG, roasted TG; RDTG, roasted–dealkalized TG; MTG-1, representative marketed TG product; QC, quality-control sample; UPLC-Q-TOF-MS/MS, ultra-performance liquid chromatography coupled with quadrupole time-of-flight tandem mass spectrometry. In panel E, solid black lines indicate the original model values. In panel F, dashed lines indicate regression lines from permutation testing.

Serum analysis was conducted to identify prototype components entering circulation post-oral administration, as these constituents may be more directly related to in vivo pharmacological and toxicological relevance than the full in vitro chemical profile [27,28]. The serum chromatogram is presented in Figure 4A. This experiment was intended to provide preliminary linkage evidence between the material chemical profile and the serum-detectable prototype profile, rather than to characterize pharmacokinetic behavior. Therefore, the serum data were interpreted only as relative UPLC-Q-TOF-MS/MS signal intensities at a single time point. A total of 63 circulating prototype constituents were annotated in rat serum, including 9 compounds confirmed by comparison with available authentic standards and 54 tentatively annotated compounds. These constituents comprised 26 diterpenoids, 16 triterpenoids, 15 alkaloids, 2 steroids, and 4 other constituents (Figure 4B and Table S3). The serum components largely matched the chemical domains identified in vitro. Of the 54 triterpenoids and 55 alkaloids detected in vitro, 16 triterpenoids and 15 alkaloids were found in serum. Additionally, 26 out of 59 diterpenoids identified in vitro entered the bloodstream, representing 41.2% of all circulating prototype constituents.

The serum profile maintained the primary chemical domains found in the material samples while showing an enrichment of constituents likely to survive gastrointestinal processing and cross biological barriers. Large and structurally complex alkaloids often exhibit limited intestinal permeability due to unfavorable physicochemical properties or transporter-mediated efflux [29,30]. Highly reactive diterpene epoxides are rapidly transformed by hepatic cytochrome P450 enzymes or conjugated to glucuronide derivatives, leading to biliary and renal excretion [31,32]. The detection of 15 alkaloid prototypes in serum suggests that these nitrogen-containing compounds can be absorbed after oral administration, despite their complex sesquiterpene pyridine frameworks, indicating their potential significance for systemic pharmacological and toxicological effects. Previous studies have demonstrated that several *Tripterygium* alkaloids, including wilforine, wilforgine, wilfortrine, and wilfordine, are detectable in plasma after oral administration and may contribute to efficacy or hepatotoxic risk [8,27,33].

Celastrol was not detected in serum under the present analytical conditions. Together with the targeted HPLC results, this suggests that celastrol-related signals were below the detection limit in processed material samples and were not detected in the corresponding single-time-point serum profiles under the present analytical conditions. Considering the known cytotoxicity and safety concerns of celastrol [34], its removal may contribute to the safety-related chemical changes associated with roasting, although direct toxicological confirmation is still required.

### 3.5. Comparison of Circulating Prototype Metabolite Profiles Among TG, RTG, and RDTG

Serum PCA and PLS-DA analyses demonstrated distinct clustering and group discrimination, as shown in Figure 4C,D. The QC samples were centered and tightly distributed, indicating stable data. RTG and RDTG were more similar to each other than to TG, suggesting that the sequential process observed in vitro was also evident at the circulating level. Cluster analysis categorized the samples into six groups (Figure 4E), aligning with the PCA and PLS-DA results. The 200-permutation test (Figure 4F) confirmed model reliability, as the permuted R2 and Q2 values were lower than the original values.

Differential screening revealed 11 circulating components distinguishing TG from RTG and 4 components differentiating RTG from RDTG, as shown in Table 3 and Table 4.

#### 3.5.1. Roasting-Related Changes in Circulating Exposure

In comparing TG and RTG, we observed that demethylzeylasteral, wilforic acid E, wilforic acid B, and triptocallic acid B increased in RTG serum, while neotriptophenolide, triptonoditerpenic acid, and triptoquinone H decreased after roasting. Demethylzeylasteral, a triterpenoid from *T. wilfordii*, has been reported to exhibit anti-inflammatory, immunomodulatory, and antitumor properties. Recent studies indicate that it alleviates inflammation and colitis by suppressing NF-κB and STAT3/5 through targeting IKKα/β and JAK2 [35]. In non-small cell lung cancer cells, it inhibits proliferation, migration, and invasion by regulating EMT-related proteins and inhibiting AKT/CREB signaling [36]. The increased serum levels of these compounds may enhance RTG’s pharmacological effects. Conversely, the serum levels of highly lipophilic constituents like neotriptophenolide, triptonoditerpenic acid, triptoquinone H, and certain triterpenoid keto acids decreased. Since neotriptophenolide and some non-specific diterpenoid lactones are linked to tissue irritation and non-selective cytotoxicity in crude drugs and extracts [37], these reductions suggest a potential decrease in toxicity-related exposure, though direct toxicological validation is needed. Overall, these results indicate that roasting alters the detectable prototype spectrum in single-time-point serum.

#### 3.5.2. Dealkalization-Related Restriction of Circulating Exposure

The comparison between RTG and RDTG involved fewer differential components but yielded a more specific process signal. Tripterygiumine T, a key alkaloid, showed a significant downregulation in RDTG serum (*p* < 0.05), consistent with the alkaloid-reducing effect of dealkalization observed in the material samples. This result suggests a reduced single-time-point serum signal of an alkaloid prototype following dealkalization. This decreased relative abundance of specific alkaloid constituents represents a reduction in known toxic chemical markers at the circulating level, though direct biological evaluation—including liver/kidney histopathology and metabolic biomarker assays—is fundamentally required to determine if this reduces real hepatorenal burden [33,38]. Additionally, the relative serum response intensities of Ejap 4, demethylzeylasteral, and wilforlide A were significantly lower in RDTG than in RTG. This finding indicates that while dealkalization may reduce exposure to alkaloids, it also decreases exposure to certain potentially active triterpenoids, suggesting a potential trade-off between efficacy and safety.

## 4. Discussion

This study redefines TG processing as a metabolite-remodeling challenge rather than merely reducing content. At the material level, roasting preserved TG’s spectral framework but significantly altered diterpenoid and triterpenoid metabolites. Celastrol became undetectable post-roasting, while wilforlide A increased significantly. At the circulating level, roasting enhanced serum responses for demethylzeylasteral, wilforic acid E, wilforic acid B, and triptocallic acid B, while reducing neotriptophenolide and triptonoditerpenic acid. These linked material–serum changes suggest that roasting modifies both extract composition and oral systemic exposure.

Dealkalization resulted in a more alkaloid-focused profile. HPLC revealed a material signature marked by the depletion of 18 alkaloid constituents and reduced wilforine content. This aligns with the acid-mediated transfer of protonated alkaloids into the aqueous phase. In serum, tripterygiumine T levels significantly decreased in RDTG, indicating reduced exposure to alkaloid-associated prototypes. However, the lower serum responses of demethylzeylasteral and wilforlide A post-dealkalization suggest that alkaloid removal might also diminish exposure to certain triterpenoid-related constituents. This finding underscores a potential chemical trade-off between active terpenoid retention and alkaloid depletion that warrants consideration during process optimization. One limitation of this study is that the discarded acidic aqueous phase was not comprehensively profiled. Although the retained organic phase showed a clear reduction in alkaloid-related constituents, the exact composition of the acid-washing fraction and any potential loss of non-alkaloid polar constituents require further investigation.

It is worth noting that the serum component of this study should be interpreted as a single-time-point, signal-based screening analysis. It provides preliminary evidence that process-induced changes in the material chemical profile are reflected, at least partly, in the serum-detectable prototype profile after oral administration. However, because serum was collected only at 1 h after the final dose and the UPLC-Q-TOF-MS/MS data were analyzed mainly as relative signal intensities, the present study cannot define pharmacokinetic parameters such as Cmax, Tmax, AUC, half-life, bioavailability, or total systemic exposure. Therefore, the observed differences in serum-detectable prototype constituents should be regarded as candidate serum linkage signals rather than definitive pharmacokinetic markers.

**Figure 5:**
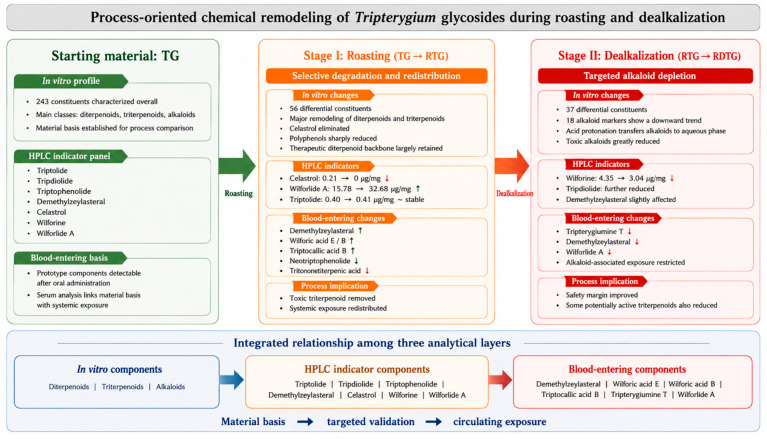
Integrated process-oriented schematic linking material metabolite remodeling, HPLC indicator validation, and the single-time-point serum-detectable prototype profile of TG during roasting and dealkalization. Arrows indicate process-induced increases or decreases among material constituents, HPLC markers, and serum-detectable prototype constituents.

A metabolite-centered quality-control strategy should integrate material markers with circulating exposure markers. The proposed process-responsive panel includes celastrol and wilforlide A as markers responsive to roasting, wilforine as a dealkalization-responsive material marker, and triptolide/tripdiolide-related diterpenoids as indicators of structural backbone preservation, which serve as candidates for future functional pharmacodynamic validation. Additionally, tripterygiumine T, demethylzeylasteral, wilforic acid E/B, and triptocallic acid B serve as serum linkage markers. This panel is not meant to establish final specifications at this stage; instead, it offers testable candidates for future pharmacokinetic, efficacy, and toxicological validation. Figure 5 summarizes the integrated relationship among material metabolite remodeling, HPLC indicator validation, and blood-entering prototype exposure.

To clarify the rationale for marker selection, the candidate process-responsive marker panel was established based on five criteria: processing responsiveness, analytical quantifiability, serum detectability at a fixed sampling time, documented toxicological or pharmacological relevance, and representativeness of chemical classes. Celastrol and wilforlide A were chosen as roasting-responsive material markers. Celastrol became undetectable after roasting, while wilforlide A exhibited a significant increase in processed samples. Both compounds were confirmed and quantified using a validated HPLC method. Wilforine was identified as a dealkalization-responsive material marker, representing the sesquiterpene pyridine alkaloid fraction, which decreased following the acid-washing step. Triptolide and tripdiolide were retained as representative diterpenoid markers due to their quantifiability by HPLC and their ability to provide information on the diterpenoid fraction affected by processing. Tripterygiumine T was selected as a serum-detectable alkaloid-related linkage marker, as its relative serum signal was lower in RDTG compared to RTG. Demethylzeylasteral, wilforic acid E, wilforic acid B, and triptocallic acid B were chosen as serum-linkage markers due to significant differences observed in the single-time-point serum prototype profile after roasting. Consequently, the proposed panel integrates material-level process responsiveness, targeted quantifiability, and serum-detectable linkage information. However, it should be considered a candidate marker panel for further validation rather than a finalized quality specification.

The chemometric screening conducted in this study was exploratory. Principal Component Analysis (PCA), Partial Least Squares Discriminant Analysis (PLS-DA), and 200-permutation testing evaluated sample separation and model robustness. Differential features were identified using a Variable Importance in Projection (VIP) threshold of ≥ 1 combined with a nominal *p*-value of ≤0.05, without applying formal false-discovery-rate correction. Consequently, the reported differential constituents should be regarded as candidate process-responsive features rather than definitive biomarkers. Future studies must involve larger sample sizes, independent validation batches, targeted quantitative assays, and false-discovery-rate-controlled statistics to confirm the robustness of these candidate markers.

## 5. Conclusions

This study highlights how roasting and dealkalization uniquely alter TG through distinct metabolite-level mechanisms by linking material metabolite profiling with serum prototype analysis. Roasting maintained the overall TG chemical structure but redistributed diterpenoid and triterpenoid components. It rendered celastrol undetectable in both material and serum profiles, increased wilforlide A in material samples, and shifted the post-dose serum profile toward demethylzeylasteral and specific wilforic/triptocallic acid derivatives. In contrast, dealkalization produced an alkaloid-focused signature, reducing wilforine at the material level and tripterygiumine T in serum while also decreasing exposure to certain triterpenoid-related constituents. These findings support a process-responsive marker panel that integrates material markers with circulating exposure markers, linking chemical processing, systemic exposure, and potential efficacy–safety trade-offs in processed botanical preparations. However, the study is limited by its focus on prototype serum constituents and pooled time-point sampling. Further pharmacokinetic, metabolite-transformation, and toxicological studies are necessary to validate these markers for process control and safety assessment.

## Figures and Tables

**Table 1:** Linear regression equations and linear ranges.

Q-Markers	Calibration Curve	r	Linear Range/(μg/mL)
Tripdiolide	y = 14,136x + 10.30	0.9993	3.82–76.37
Triptolide	y = 19,461x − 7.73	0.9998	8.22–164.40
Triptophenolide	y = 2671.3x + 1.56	1.0000	13.67–273.32
Demethylzeylasteral	y = 7168.6x − 17.46	0.9996	5.84–187.00
Wilforine	y = 6325.3x + 3.21	0.9998	4.98–79.74
Celastrol	y = 7759.1x − 1.96	0.9999	8.06–51.00
Wilforlide A	y = 3032.2x − 26.97	0.9990	32.30–100.93

**Table 2:** Contents of seven representative HPLC marker constituents in TG-related samples (*n* = 3).

Sample	Content/(μg/mg)						
	Tripdiolide	Triptolide	Triptophenolide	Demethylzeylasteral	Wilforine	Celastrol	Wilforlide A
TG	0.82 ± 0.07	0.40 ± 0.19	12.27 ± 0.32	4.60 ± 0.03	4.12 ± 0.82	0.21 ± 0.09	15.78 ± 0.20
RTG	0.17 ± 0.01 ***	0.41 ± 0.06	11.83 ± 0.59	3.02 ± 0.04 ***	4.35 ± 0.03	ND	32.68 ± 0.45 *
RDTG	0.18 ± 0.00 ***	0.46 ± 0.03	13.49 ± 0.09 ***	4.11 ± 0.02 ^##^	3.04 ± 0.01 ** ^##^	ND	38.17 ± 0.94 ***
MTG1	1.79 ± 0.05 *** ^###^	0.30 ± 0.16	2.69 ± 0.03 *** ^###^	76.71 ± 0.14 *** ^###^	1.96 ± 0.02 *** ^###^	3.98 ± 0.24 *** ^###^	8.36 ± 0.09 ^###^
MTG2	0.40 ± 0.06 *** ^##^	0.10 ± 0.02	6.62 ± 0.06 *** ^###^	1.35 ± 0.03 *** ^###^	5.78 ± 0.19 ** ^##^	0.23 ± 0.00	28.06 ± 1.80
MTG3	0.51 ± 0.01 *** ^###^	0.83 ± 0.33	3.06 ± 0.02 *** ^###^	14.55 ± 0.56 *** ^###^	8.69 ± 0.10 *** ^###^	4.09 ± 0.33 *** ^###^	23.56 ± 3.89
MTG4	1.33 ± 0.05 *** ^###^	2.16 ± 0.40 *** ^###^	6.08 ± 0.01 *** ^###^	13.31 ± 0.01 *** ^###^	13.08 ± 0.15 *** ^###^	3.40 ± 0.01 *** ^###^	11.83 ± 0.12 ^##^
MTG5	0.91 ± 0.03 ^###^	0.82 ± 0.20	8.83 ± 1.57 *** ^##^	20.42 ± 0.39 *** ^###^	12.14 ± 0.04 *** ^###^	4.97 ± 0.02 *** ^###^	13.19 ± 0.23 ^##^

Note: Data are presented as mean ± SD. TG, self-prepared *Tripterygium* glycosides; RTG, roasted TG; RDTG, roasted–dealkalized TG; MTG1–MTG5, marketed *Tripterygium* glycoside products; ND, not detected; SD, standard deviation. Compared with TG, * *p* < 0.05, ** *p* < 0.01, *** *p* < 0.001; compared with RTG, ^##^ *p* < 0.01, ^###^ *p* < 0.001.

**Table 3:** Candidate differential serum-detectable prototype constituents between TG and RTG.

NO.	Compound Name	Molecular Formula	Category	RTG vs. TG		
				VIP Value	*p* Value	Trend
1	Neotriptophenolide	C_21_H_26_O_4_	Diterpenoids	1.21	0.00	↓
2	Triptonoditerpenic acid	C_21_H_28_O_4_	Diterpenoids	1.09	0.01	↓
3	Triptoquinone H	C_20_H_26_O_3_	Diterpenoids	1.05	0.02	↓
4	Triptotriterpenic acid A	C_30_H_48_O_4_	Triterpenoids	1.65	0.00	↓
5	Demethylzeylasteral	C_29_H_36_O_6_	Triterpenoids	1.26	0.02	↑
6	Orthosphenic acid	C_30_H_48_O_5_	Triterpenoids	1.23	0.00	↓
7	Triptotriterpenonic acid A	C_30_H_46_O_4_	Triterpenoids	1.68	0.00	↓
8	Wilforic acid E	C_30_H_46_O_5_	Others	2.02	0.00	↑
9	Wilforic acid B	C_29_H_44_O_4_	Others	1.03	0.03	↑
10	Triptocallic acid B	C_30_H_48_O_3_	Triterpenoids	1.16	0.00	↑
11	Ent-pimara-8(14),15-diene-19-ol	C_20_H_32_O	Triterpenoids	1.62	0.00	↑

Note: TG, self-prepared *Tripterygium* glycosides; RTG, roasted TG; VIP, variable importance in projection. Candidate differential constituents were screened using VIP ≥ 1 and nominal *p* ≤ 0.05. ↑ indicates increased relative response intensity in RTG compared with TG; ↓ indicates decreased relative response intensity in RTG compared with TG.

**Table 4:** Candidate differential serum-detectable prototype constituents between RTG and RDTG.

NO.	Compound Name	Molecular Formula	Category	RDTG vs. RTG	
				VIP Value	*p* Value
1	Tripterygiumine T	C_32_H_39_NO_16_	Alkaloids	1.84	0.02
2	Ejap 4	C_32_H_40_O_13_	Triterpenoids	1.79	0.02
3	Demethylzeylasteral	C_29_H_36_O_6_	Triterpenoids	1.30	0.01
4	Wilforlide A	C_30_H_46_O_3_	Triterpenoids	1.18	0.00

Note: RTG, roasted *Tripterygium* glycosides; RDTG, roasted–dealkalized TG; VIP, variable importance in projection. Candidate differential constituents were screened using VIP ≥ 1 and nominal *p* ≤ 0.05. All listed constituents showed decreased relative UPLC-Q-TOF-MS/MS response intensity in RDTG compared with RTG.

## Data Availability

The data presented in this study are available in the article and Supplementary Materials. Additional raw data are available from the corresponding author upon reasonable request.

## Supplementary Materials

The following supporting information can be downloaded at https://www.mdpi.com/article/10.3390/metabo16070476/s1.
