# Supplementary material for: Process-Induced Metabolite Remodeling of Tripterygium Glycosides and Its Association with Circulating Prototype Constituents
Source: Metabolites. 2026 Jul 7;16(7):476. doi: 10.3390/metabo16070476 (PMC13413455; doi:10.3390/metabo16070476)
Supplement: Supplementary file 1 [file metabolites-16-00476-s001.zip › metabolites-4360233-supplementary.pdf]

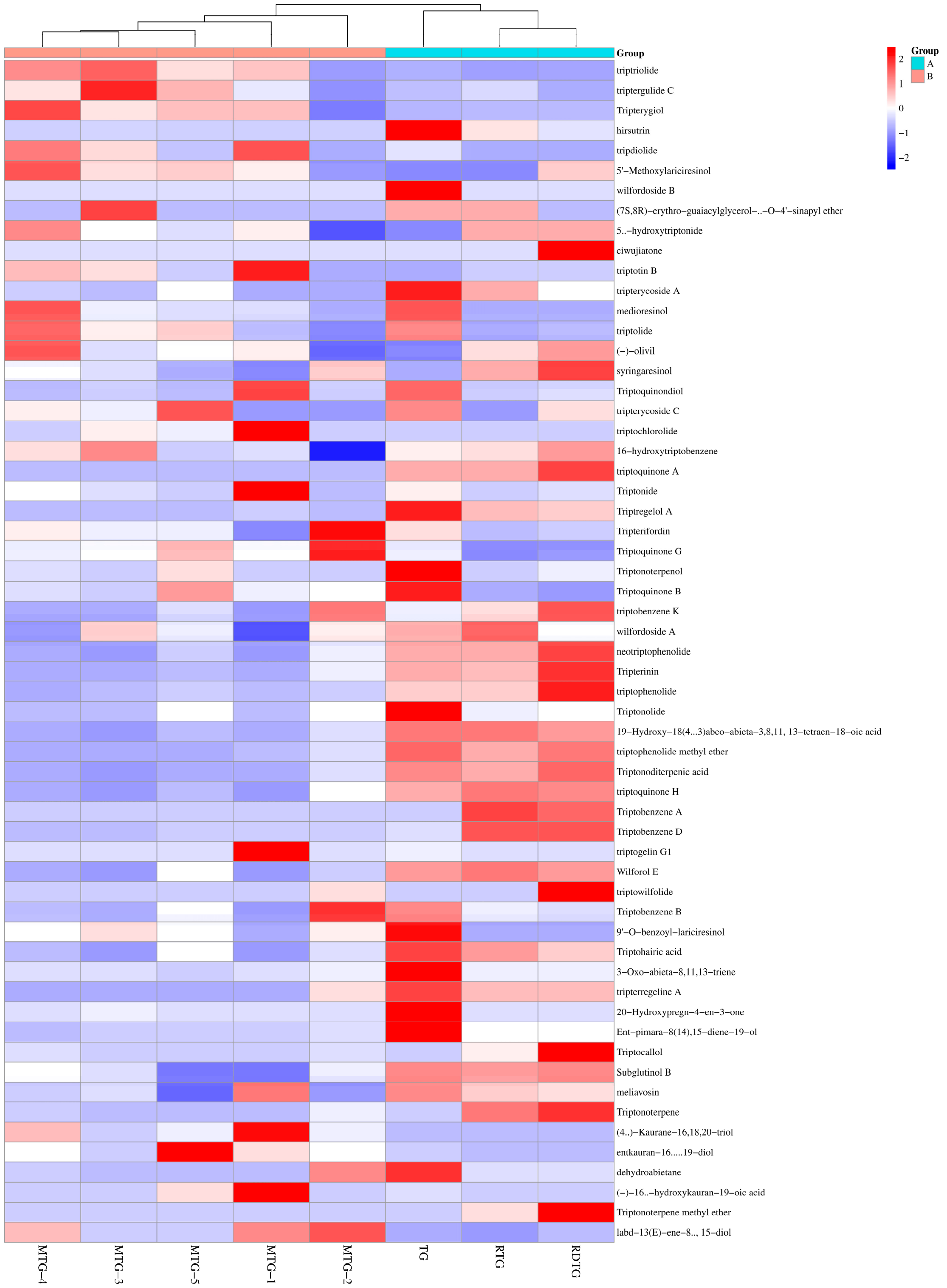

Figure S1. Heatmaps of diterpenoids in TG-related material samples. The heatmap color scale represents normalized relative UPLC-Q-TOF-MS/MS response intensity. TG, self-prepared *Tripterygium* glycosides; RTG, roasted TG; RDTG, roasted-dealkalized TG; MTG1 – MTG5, marketed *Tripterygium* glycoside products.

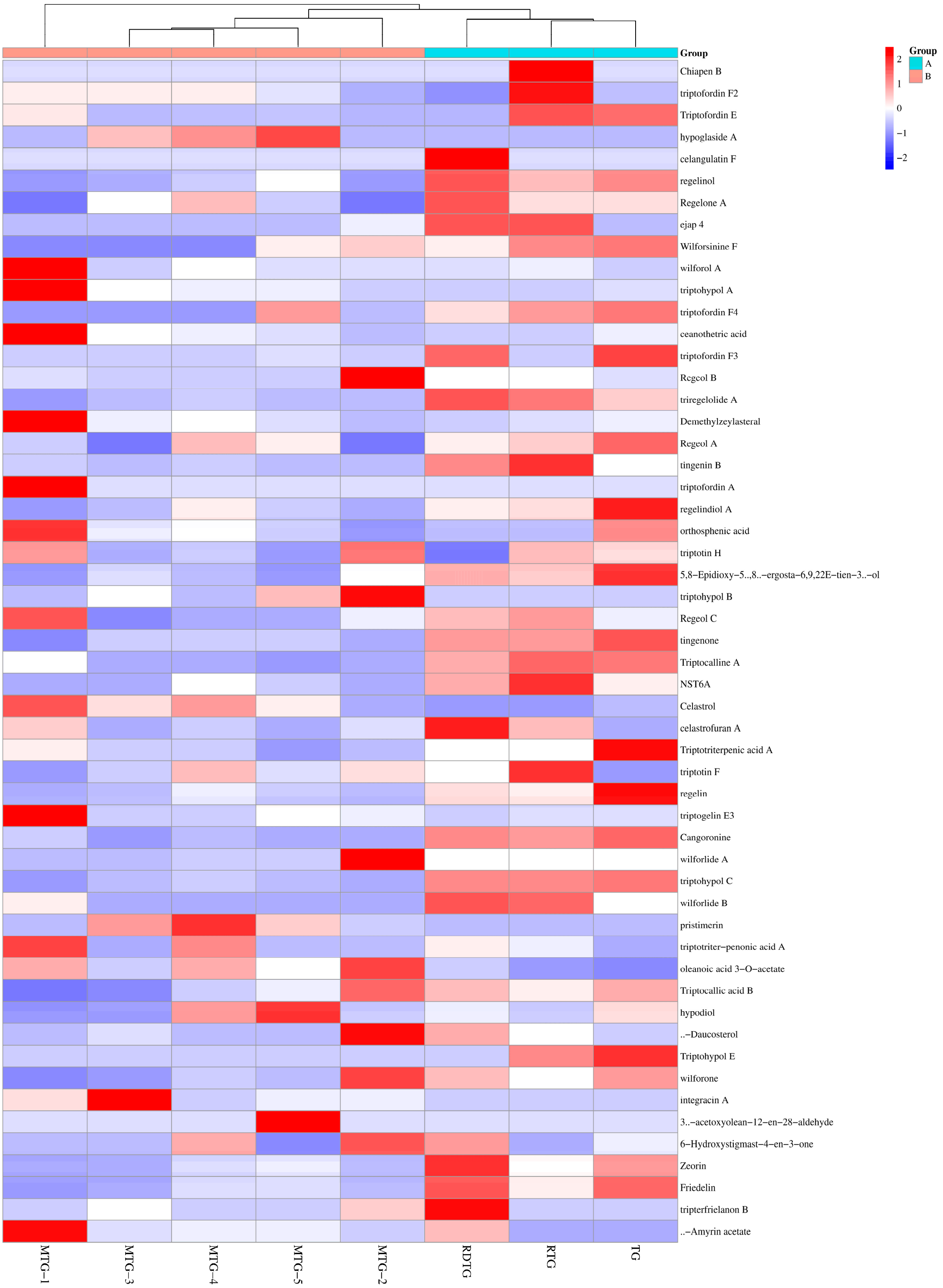

Figure S2. Heatmaps of triterpenoids in TG-related material samples. The heatmap color scale represents normalized relative UPLC-Q-TOF-MS/MS response intensity. TG, self-prepared *Tripterygium* glycosides; RTG, roasted TG; RDTG, roasted-dealkalized TG; MTG1 − MTG5, marketed *Tripterygium* glycoside products.

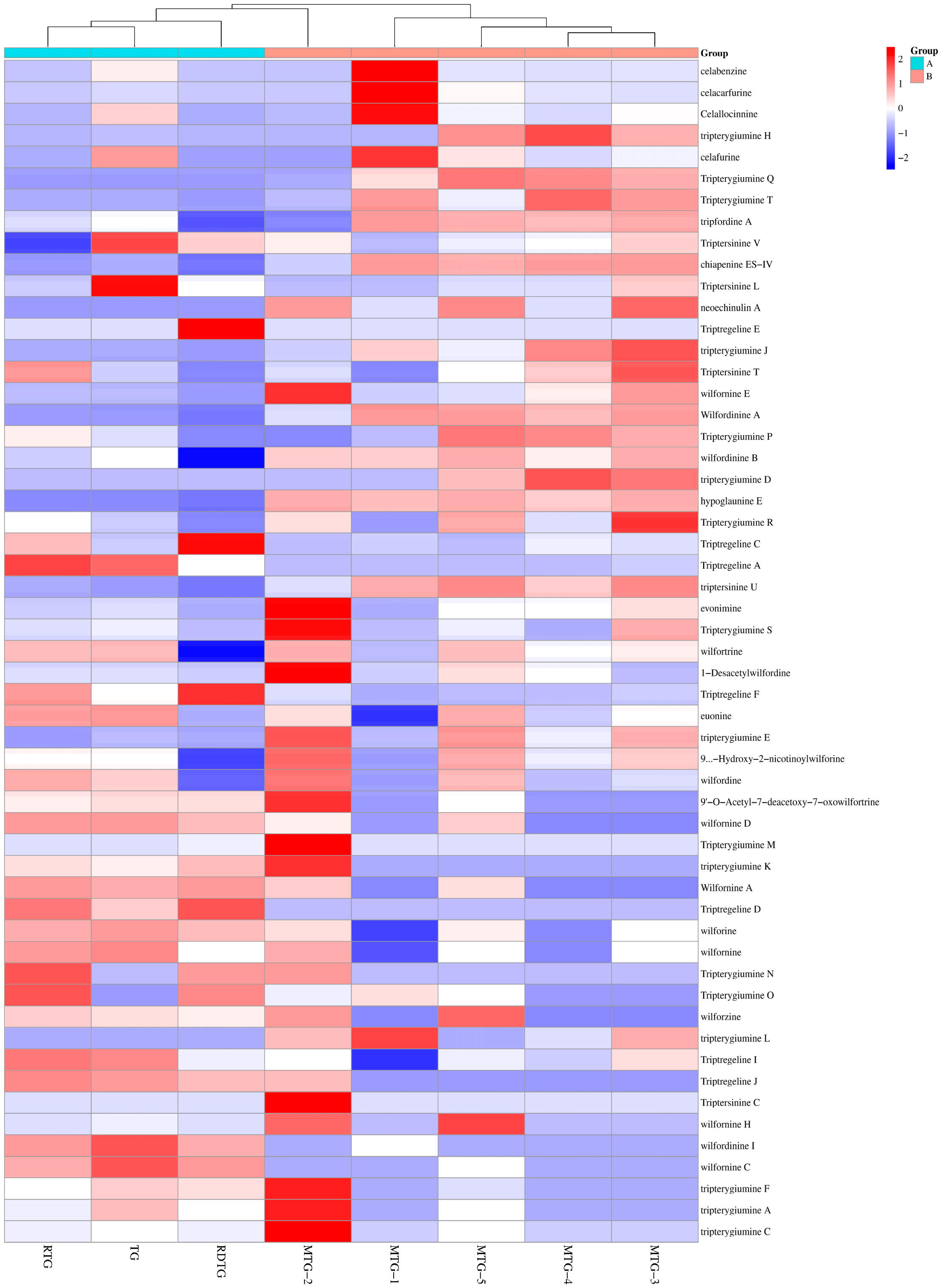

Figure S3. Heatmaps of alkaloids in TG-related material samples. The heatmap color scale represents normalized relative UPLC-Q-TOF-MS/MS response intensity. TG, self-prepared *Tripterygium* glycosides; RTG, roasted TG; RDTG, roasted-dealkalized TG; MTG1 – MTG5, marketed *Tripterygium* glycoside products.

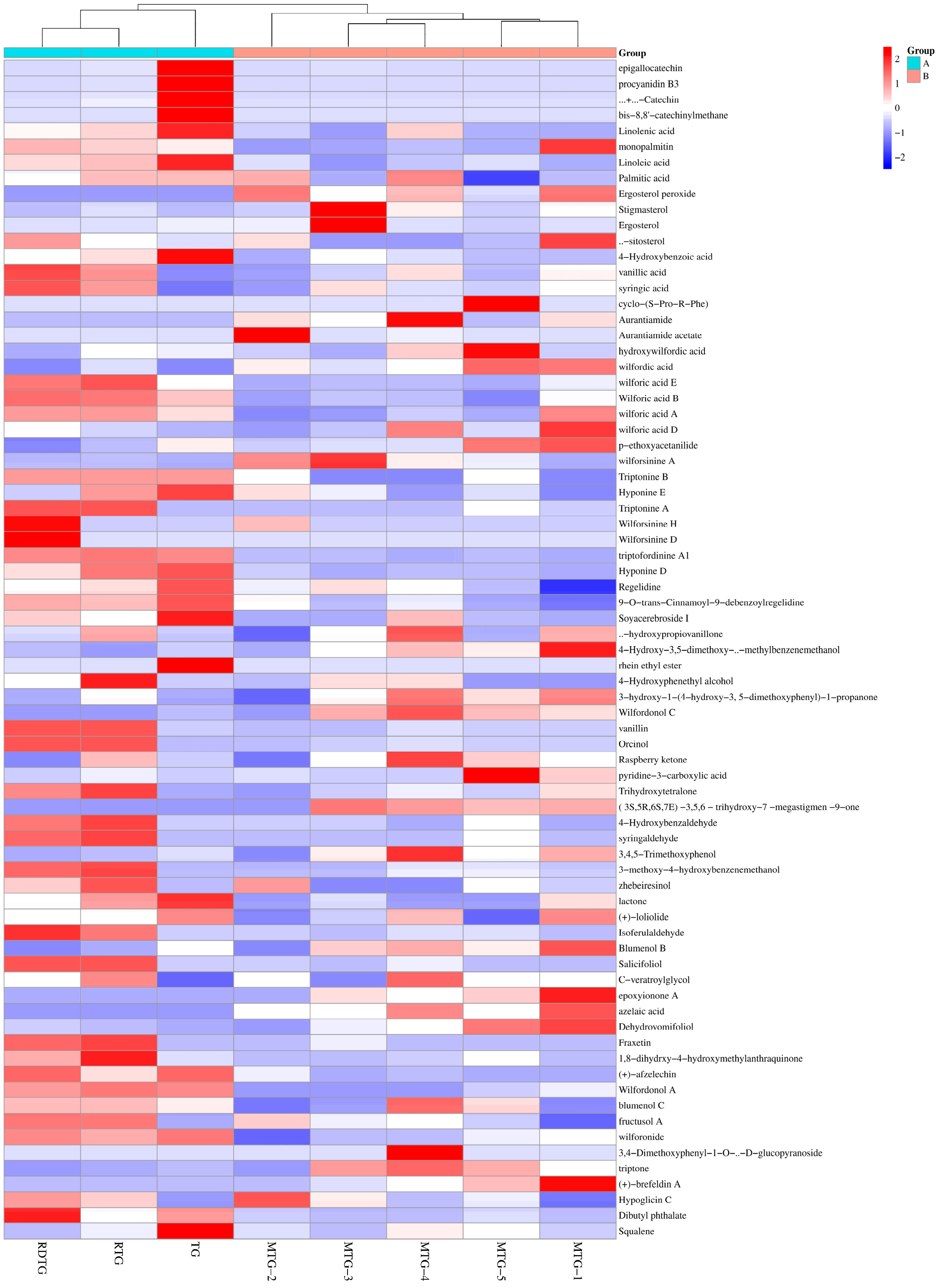

Figure S4. Heatmaps of other constituents in TG-related material samples. The heatmap color scale represents normalized relative UPLC-Q-TOF-MS/MS response intensity. TG, self-prepared *Tripterygium* glycosides; RTG, roasted TG; RDTG, roasted-dealkalized TG; MTG1 – MTG5, marketed *Tripterygium* glycoside products.

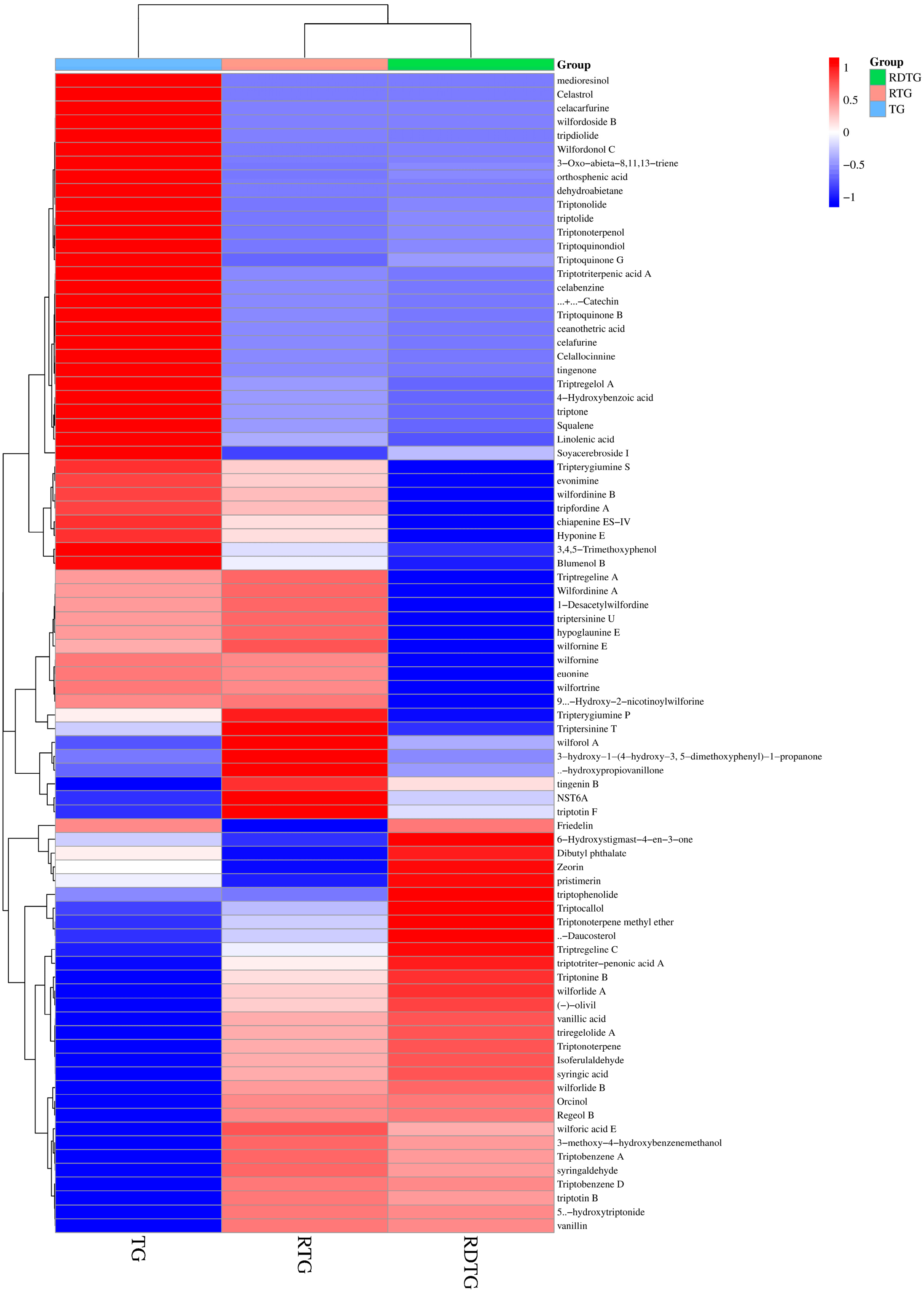

Figure S5. Heatmap of candidate differential constituents between TG and RTG and between RTG and RDTG. Candidate differential constituents were screened using VIP $\geqslant$ 1 and nominal P $\leqslant$ 0.05. The heatmap color scale represents normalized relative UPLC-Q-TOF-MS/MS response intensity. TG, self-prepared *Tripterygium* glycosides; RTG, roasted TG; RDTG, roasted-dealkalized TG; VIP, variable importance in projection.

Table S1. Annotated constituents detected in TG-related material samples.

NO.	T_R/min	Compounds	Molecular formula	Mode	Measured mass (m/z)	ppm	Fragment ions	Category	Source
1	0.83	hydroxywilfordic acid	C11H13NO5	+H	240.08614	-2.1	240.0870、222.0764、204.0649、194.0807、176.0704、148.0755、134.0595、117.0593、106.0655、92.0525、78.0372	Others	a, b, d, e, g, h
2	0.87	wilfordic acid	C11H13NO4	+H	224.09113	-2.7	224.0947、206.0814、178.0869、160.0757、132.0814、108.0440、92.0514、80.0512	Others	b, d, e, f, g, h
3	0.99	epigallocatechin	C15H14O7	+H	307.08107	-0.5	307.0817、181.0496、163.0389、139.0388、127.0391、107.0504	Polyphenols	a, b, c, f
4	1.99	4-Hydroxybenzoic acid	C7H6O3	+H	139.03909	0.9	139.0393、121.0318、111.0445、93.0348、77.0409、69.0002	Amides	a, b, c, d, e, f, g, h
5	2.08	procyanidin B3	C30H26O12	+H	579.14944	-0.5	579.1595、427.1001、409.0923、301.0704、289.0709、275.0555、247.0623、139.0399、127.0402、123.0438	Polyphenols	a,b
6	2.37	vanillic acid	C8H8O4	+H	169.04959	0.3	169.0497、151.0416、123.0442、111.0053、109.0303、95.0498、69.0005、55.0201	Amides	a, b, c, d, e, f, g, h
7	2.38	β-hydroxypropiovanillone	C10H12O4	+H	197.08077	-0.3	197.0823、179.0703、151.0396、139.0385、123.0452、93.0366、73.0327、55.0245	Others	a, b, c, d, e, f, g, h
8	2.56	syringic acid	C9H10O5	+H	199.06	-0.5	199.0673、181.0496、155.0704、125.0238、121.0297、69.0000、55.0231	Amides	a, b, c, d, e, f, g, h
9	2.6	4-Hydroxy-3,5-dimethoxy-α-methylbenzenemethanol	C10H14O4	+H	199.09616	-1.6	199.1009、181.0861、167.0353、155.0715、137.0598、125.0246、121.0661、55.0238	Others	a, c, d, e, f, g, h

10	2.69	(+)-Catechin	C15H14O6	+H	291.08607	-0.8	291.0858、207.0468、165.0543、139.0387、123.0444、111.0452、95.0510、83.0512	Polyphenols	a, b, c, f
11	2.7	rhein ethyl ester	C17H12O6	+H	313.06911	-5	313.0701	Others	a
12	2.93	4-Hydroxyphenethyl alcohol	C8H10O2	+H	139.07505	-2.2	139.0752、121.0582、91.0560、77.0417、	Others	a, b, c, e, f, g
13	2.94	3-hydroxy-1-(4-hydroxy-3,5-dimethoxyphenyl)-1-propanone	C11H14O5	+H	227.09132	-0.4	227.0912、209.0817、181.0500、167.0704、155.0709、123.0452、73.0322、55.0242	Others	a, b, c, d, f, g, h
14	3.09	celafurine	C21H27N3O3	+H	370.21215	-1	370.2113、266.1491、249.1228、223.1447、166.0859、160.1112、112.1128、100.0761、95.0136、91.0555、72.0837	Alkaloids	a, b, c, d, e, f, g, h
15	3.36	Wilfordonol C	C13H20O3	+H	225.14812	-1.8	225.1480、207.1386、195.1376、189.1275、183.1384、177.1282、165.1282、159.1181、151.1125、149.0973、137.0965、123.0812、99.0463、71.0533、55.0609	Others	a, b, c, d, e, f, g, h
16	3.52	vanillin	C8H8O3	+H	153.05477	1	153.0546、125.0606、109.0291、93.0354	Others	a, b, c, d, e, f, g, h
17	3.63	Orcinol	C7H8O2	+H	125.05979	0.7	125.0608、111.0461、93.0357、81.0384	Others	a, b, c, d, e, f, g, h
18	3.66	Raspberry ketone	C10H12O2	+H	165.09094	-0.4	165.0907、147.0814、131.0502、123.0812、107.0504、105.0703、95.0526、91.0560、77.0419、55.0204	Others	a, b, c, d, e, f, g, h
19	3.69	pyridine-3-carboxylic acid	C6H5NO2	+H	124.03969	3.1	124.0398、106.0298、80.0513、78.0360、52.0224	Others	b, d, e, h
20	3.71	triptriolide	C20H26O7	+H	379.17525	0.3	379.1740、227.1067、213.0903、167.0841、149.0601、133.0655、105.0706、71.0520	Diterpenoids	a, b, c, d, e, f, g, h

21	3.76	Trihydroxytetralone	C10H10O4	+H	195.065	-0.9	195.0652、177.0549、163.0371、153.0569、149.0605、107.0494、89.0408、55.0232	Others	a, b, c, d, e, f, g, h
22	3.77	(3S,5R,6S,7E) -3,5,6 -trihydroxy-7 -megastigmen -9-one	C13H22O4	+H	243.15895	-0.6	243.1602、225.1464、207.1363、189.1285、185.1183、161.1328、147.1186、105.0706、91.0559、69.0355、55.0238	Others	b, c, d, e, f, g, h
23	3.97	bis-8,8'-catechinylmethane	C31H28O12	+H	593.16492	-0.7	593.1294、575.1182、453.0840、441.0812、427.0963、305.1037、291.0863、287.0651、137.0595、123.0422	Polyphenols	a
24	4.12	4-Hydroxybenzaldehyde	C7H6O2	+H	123.04415	0.7	123.0467、105.0335、95.0522、77.0419、55.0224	Others	a, b, d, e, f, h
25	4.17	syringaldehyde	C9H10O4	+H	183.06501	-0.9	183.0658、165.0539、155.0706、125.0234、123.0448、53.0445	Others	b, c, h
26	4.25	3,4,5-Trimethoxyphenol	C9H12O4	+H	185.08064	-1	185.0806、169.0496、153.0545、139.0391、127.0394、125.0602、99.0464、59.0189	Others	a, b, c, d, e, f, g, h
27	4.29	3-methoxy-4-hydroxybenzenemethanol	C8H10O3	+H	155.07012	-1	155.0700、125.0230、123.0445、98.9851、97.0301、95.0508、55.0221	Others	a, b, c, d, e, f, g, h
28	4.44	triptergulide C	C20H28O8	+H	397.18547	-0.6	397.1869、379.1771、361.1723、315.1585、297.1482、265.1201、247.1003、167.0912、151.0785、131.0860	Diterpenoids	a, b, c, d, e, f, g, h
29	4.53	zhebeiresinol	C14H16O6	+H	281.1017	-1	281.1034、263.0895、231.0643、221.0800、219.0659、185.0769、167.0685、141.0691、127.0404、115.0553、55.0243	Others	a, b, c, d, e, h
30	4.92	lactone	C14H12O3	+H	229.08568	-1.1	229.0853、211.0754、201.0926、183.0802、143.0852、79.0573、55.0276	Others	a, b, c, d, f

31	5.33	cyclo-(S-Pro-R-Phe)	$C_{14}H_{16}N_2O_2$	+H	245.12844	-0.1	245.1288、217.1320、200.1067、153.0669、120.0813、98.0602、70.0676	Phenolic compounds	h
32[a]	5.37	celabenzine*	$C_{23}H_{29}N_3O_2$	+H	380.23308	-0.5	380.2325、281.1657、275.1757、259.1444、188.1072、176.1067、160.1112、131.0505、105.0342、100.0770、91.0563、72.0845	Alkaloids	a, b, d, e, f, g, h
33	5.56	Tripterygiol	$C_{22}H_{28}O_8$	+H	421.18488	-1.9	421.1788、371.1519、267.1260、249.1123、219.1040、199.0756、167.0706、131.0859、103.0511	Diterpenoids	a, b, c, d, f, g, h
34	5.57	(+)-loliolide	$C_{11}H_{16}O_3$	+H	197.11714	-0.4	197.1172、179.1066、167.1113、151.1098、137.0968、113.0592、103.0552、95.0514、59.0532、55.0596	Others	a, b, c, d, e, f, g, h
35	5.71	hirsutrin	$C_{21}H_{20}O_{12}$	+H	465.10246	-0.6	465.1105、303.0509、285.0376、163.0396、127.0415、111.0096、85.0309	Diterpenoids	a, b, c, f
36[a]	5.97	tripdiolide*	$C_{20}H_{24}O_7$	+H	377.15976	0.7	377.1595、359.1476、341.1376、331.1557、297.1137、291.0841、267.1374、263.0917、253.1217、213.0914、199.0745、169.1011、129.0715、117.0724、85.0660	Diterpenoids	a, b, c, d, e, f, g, h
37	5.98	p-ethoxyacetanilide	$C_{10}H_{13}NO_2$	+H	180.1017	-1.2	180.1029、152.0711、134.0608、108.0457、93.0591、92.0506、79.0574	Others	a, b, c, d, e, f, g, h
38	6.07	Isoferulaldehyde	$C_{10}H_{10}O_3$	+H	179.07013	-0.8	179.0693、161.0594、147.0434、135.0436、133.0649、119.0495、107.0505、91.0561、79.0570、55.0237	Others	a, b, c, d, e, f, g, h

39	6.22	5'-Methoxylariciresinol	C21H26O7	+H	391.17508	-0.1	391.1828、373.1345、341.0993、251.0678、237.0822、225.0885、219.1017、205.0844、181.0672、167.0680、153.0549、137.0596、121.0664、113.0630	Diterpenoids	b, c, d, e, f, g, h
40	6.24	Blumenol B	C13H22O3	+H	227.16398	-0.9	227.1693、209.1541、191.1429、173.1331、165.1273、153.0910、147.1168、137.0963、125.0976、105.0712、83.0515、67.0583、55.0594	Others	a, b, d, f, g, h
41	6.45	Salicifoliol	C13H14O5	+H	251.09029	-4.4	251.0921、233.0773、219.0643、201.0556、191.0716、177.0561、135.0450、107.0512、93.0702	Others	a, b, c, e, g
42 [a]	6.89	celacarfurine*	C21H25N3O4	+H	384.192	0.6	384.1909、367.1661、299.1405、280.1291、254.1582、237.1240、219.1130、207.0768、204.1019、193.0978、188.1072、181.0970、169.0977、152.0708、131.0496、107.0504、95.0141、86.0624、56.0554	Alkaloids	a, d, f, g, h
43	7.15	C-veratroylglycol	C10H12O5	+H	213.07568	-0.3	213.0712、181.0414、167.0701、149.0066、123.0447、107.0500、95.0499	Others	a, b, c, d, e, f, g, h
44	7.34	epoxyionone A	C19H28O6	+H	353.19579	-0.2	353.1941、335.1831、281.1827、263.1255、235.1287、229.1214、215.1077、199.1108、185.0958、169.0992、137.0601、71.0511	Others	d, f, g, h
45	7.77	azelaic acid	C9H16O4	+H	189.11202	-0.6	189.1230、171.0971、125.0983、117.0719、97.1022、83.0882、69.0750	Others	d, e, f, g, h

46	7.78	Dehydrovomifoliol	C13H18O3	+H	223.13271	-0.7	223.1335、205.1231、165.0914、157.1010、153.0901、135.1176、125.0617、81.0729、69.0738、53.0442	Others	a, b, c, d, e, f, g, h
47	7.84	wilfordoside B	C26H38O9	+H	495.2586	-0.5	495.2611、333.2061、315.1961、297.1846、285.1849、165.0917、85.0297、69.0368	Diterpenoids	a
48 [a]	8.13	Celallocinnine*	C25H31N3O2	+H	406.24913	0.6	406.2480、302.1870、276.2066、202.1231、188.1074、160.1115、131.0494、103.0556、100.0773、91.0565	Alkaloids	a, b, c, d, e, f, g, h
49	8.27	Fraxetin	C10H8O5	+H	209.04363	-3.9	209.0440、181.0443、163.0339、137.0223、68.9982、55.0277	Others	b, c, f
50	8.34	(7S,8R)-erythro-guaiacylglycerol-β-O-4'-sinapyl ether	C21H26O8	+H	407.16972	-0.8	407.1617、389.0628、235.0957、197.0869、193.0895、175.0833、167.0696、161.0598、151.0368、137.0623、123.0438、81.0328	Diterpenoids	a, b, f
51	8.46	1,8-dihydrxy-4-hydroxymethylanthraquinone	C15H10O5	+H	271.06004	-0.2	271.0630、253.0567、243.0679、225.0564、181.0645、153.0708、149.0210、123.0445、121.0273、95.0517	Others	a, b, c, g, h
52	8.54	5α-hydroxytriptonide	C20H22O7	+H	375.14377	-0.2	375.1467、357.1317、295.0989、279.1025、267.1033、211.1492、165.0550、81.0371	Diterpenoids	a, b, c, d, e, f, g, h
53	8.92	ciwujiatone	C22H26O9	+H	435.16456	-0.9	435.1647、417.1533、399.1417、387.1447、281.1000、263.0907、251.0916、235.0958、221.0811、181.0499、167.0702、123.0451	Diterpenoids	c
54	9.39	(+)-afzelechin	C15H14O5	+H	275.09116	-0.9	275.0905、151.0384、123.0453、77.0405	Others	a, b, c, d, e, f, g, h
55	9.59	triptotin B	C20H26O6	+H	363.18037	0.4	363.1793、345.1695、327.1591、303.1219、267.1018、95.0864	Diterpenoids	a, b, c, d, e, f, g, h

56 [a]	9.85	tripterycoside A	C26H34O9	+H	491.22711	-0.9	491.2274、329.1741、311.1640、287.1285、165.0912、109.0299、85.0304、69.0370	Diterpenoids	a, b, c, d, f, g, h
57	10.02	medioresinol	C21H24O7	+H	389.15916	-0.8	389.3108、339.1237、267.1001、249.0910、221.0969、205.0858、193.0860、167.0700、123.0440、115.0562、91.0566	Diterpenoids	a, d, f, g, h
58	10.87	Chiapen B	C37H42O13	+H	695.27034	0.7	695.2463、667.2408、635.2572、579.2134、575.2462、537.2195、159.0565、135.0669	Triterpenoids	b
59	11.16	triptolide*	C20H24O6	+H	361.16467	0.3	361.1636、251.1441、213.0925、199.1126、167.0854、153.0706、117.0712、109.0667、97.0662、85.0677、81.0725	Diterpenoids	a, b, c, d, e, f, g, h
60	11.25	tripterygiumine H	C28H37NO13	+H	596.23332	-0.7	596.2305、578.2227、560.2127、536.2126、518.2023、224.0925、206.0814、178.0866、160.0762、132.0821	Alkaloids	a, f, g, h
61	11.36	Wilfordonol A	C13H24O3	+H	229.17982	0	229.1579、211.1051、193.1580、175.1484、149.0937、141.0688、123.1180、71.0507、55.0643	Others	a, b, c, d, h
62	11.54	(-)-olivil	C20H20O7	+H	373.12785	-0.9	343.1574、327.1190、313.1034、253.0947、239.0792、221.0846、207.0658、195.0673、179.0733、153.0548、135.0443、125.0596、107.0513、71.0156	Diterpenoids	a, b, c, d, e, f, g, h
63	11.56	Tripterygiumine Q	C33H39NO16	+H	706.23384	-0.5	706.2310、688.2256、576.2073、213.0908、152.0714、134.0614	Alkaloids	d, e, f, g, h
64	11.69	blumenol C	C13H22O2	+H	211.16926	0	211.1660、193.1603、167.0709、153.1254、137.0590、125.0988、85.0681、81.0716、71.0532、55.0637	Others	a, b, c, d, e, f, g, h

65	11.98	Tripterygiumine T	$C_{32}H_{39}NO_{16}$	+H	694.23348	-1	694.2303、676.2195、666.2348、658.2130、648.2275、616.2025、250.1068、222.0756、204.0641、194.0798、176.0695、148.0756、134.0602	Alkaloids	a, b, d, e, f, g, h
66	12.19	syringaresinol	$C_{22}H_{26}O_{8}$	+H	419.16974	-0.7	419.1701、401.1600、383.1490、371.1485、265.1068、247.0963、235.0967、167.0703、123.0447、115.0564、55.0233	Diterpenoids	a, b, c, d, e, f, g, h
67	12.22	fructusol A	$C_{13}H_{14}O_{4}$	+H	235.09634	-0.6	235.0902、217.0842、205.0800、187.0729、175.0753、173.0591、161.0613、147.0432、81.0349	Others	a, b, c, d, e, f, g, h
68	12.59	wilforonide	$C_{13}H_{16}O_{3}$	+H	221.11702	-0.9	221.1174、203.1045、179.1048、175.1124、161.0974、151.0736、137.0938、123.0816、109.0646、69.0371、55.0259	Others	a, b, c, d, e, f, g, h
69	12.6	triptofordin F2	$C_{35}H_{40}O_{13}$	+H	669.25473	0.8	669.2522、651.2520、627.2306、609.2335、549.2296、201.0810、135.0634	Triterpenoids	a, b, d, e, f, g, h
70	12.93	triptochlorolide	$C_{20}H_{25}ClO_{6}$	+H	397.14132	0.2	397.1407、341.2438、245.0711、167.0833、121.0634、113.1088、105.0712、83.0502	Diterpenoids	d, f, h
71 [a]	14.17	tripfordine A*	$C_{36}H_{45}NO_{18}$	+H	780.27058	-0.5	780.2682、762.2566、752.2729、744.2482、734.2625、720.2505、710.2669、692.2527、660.2298、250.1069、236.0919、222.0759、204.0467、194.0800、176.0696、134.0604	Alkaloids	a, b, c, d, e, f, g, h
72	14.41	Triptofordin E	$C_{35}H_{38}O_{13}$	+H	667.23889	0.5	667.2321、649.2277、607.2200、515.1504、441.1710、219.0671、163.0369、137.0578	Triterpenoids	a, b, c, d, e, f, g, h

73	14.47	Triptersinine V	C33H38O12	+H	627.24315	-0.7	627.2423、609.2305、577.1903、549.2350、475.1427、399.1496、385.1285、235.0967、191.0715、149.0587、125.0572	Alkaloids	a, c, d, e, f, g, h
74	14.5	chiapenine ES-IV	C34H41NO17	+H	736.24405	-0.9	736.2389、718.2280、708.2440、700.2212、690.2346、676.2204、658.2093、634.2116、606.2144、250.1059、222.0755、204.0640、194.0797、176.0693、148.0758、134.0604	Alkaloids	a, b, d, e, f, g, h
75	14.77	Triptersinine L	C32H41NO12	+H	632.2696	-0.9	632.2698、614.2553、579.2367、183.0594、147.0836、134.0350、124.0435、106.0403	Alkaloids	a, b, c, d, e, f, g, h
76	14.94	Triptoquinondiol	C20H28O4	+H	333.20595	-0.3	333.2056、257.1540、243.1385、231.1376、217.1218、207.1014、187.0747、175.0752、163.0752、149.0597、97.0667	Diterpenoids	a, b, c, d, e, f, g, h
77	15.52	3,4-Dimethoxyphenyl-1-O-β-D-glucopyranoside	C14H20O8	+H	317.12459	4.7	317.1258、169.1017、153.0712、141.0711、123.0832、105.0354、77.0419	Others	g
78	15.64	neoechinulin A	C19H21N3O2	+H	324.17072	0.2	324.1706、268.1087、256.1077、255.1004、240.0771、238.0978、212.1164、168.0789、156.0689、139.0498、69.0733	Alkaloids	d, e, f, g, h
79	16.54	tripterycoside C	C26H32O10	+H	505.20634	-1	505.2061、445.1503、385.1656、343.1542、325.1456、299.0911、283.0957、145.0492、109.0279、85.0309	Diterpenoids	a, c, f, g, h

80	16.67	Triptregeline E	C35H37NO12	+H	664.23685	-3	664.2344、646.2079、604.1933、587.1889、545.1783、527.1670、525.1931、485.1563、467.1460、423.1580、272.1001、137.0561、123.0441	Alkaloids	c
81	16.81	tripterygiumine J	C34H43NO16	+H	722.26487	-0.8	722.2608、704.2518、662.2438、644.2325、224.0924、206.0798、178.0853、160.0761、132.0820	Alkaloids	a, b, c, d, e, f, g, h
82	17.23	triptone	C12H18O2	+H	195.13807	0.6	195.1369、165.0880、151.1138、123.0827、83.0521、69.0738、57.0380	Others	a, b, c, d, f, g, h
83	17.64	16-hydroxytriptobenzene H	C21H28O5	+H	361.20063	-0.9	361.1726、343.1927、325.1813、315.1917、301.1439、299.2003、283.1329、275.1324、269.1236、257.1548、209.1178、195.1034、181.0955、165.0897、151.0746	Diterpenoids	a, b, c, d, e, f, g, h
84	17.88	Triptersinine T	C27H35NO10	+H	534.23316	-0.4	534.2317、516.2275、474.2030、432.2068、414.1906、351.1838、131.0845、124.0403、106.0311、78.0358	Alkaloids	a, b, e, f, g, h
85	18.1	wilfornine E	C36H43NO18	+H	778.25476	-0.7	778.2517、760.2400、750.2559、732.2469、718.2327、690.2365、648.2266、250.1068、222.0761、204.0468、176.0695、148.0757	Alkaloids	a, b, d, e, f, g, h
86	18.18	Wilfordinine A	C36H45NO17	+H	764.27473	-1.7	764.2707、746.2603、728.2517、704.2504、686.2399、662.2437、644.2305、224.0923、206.0806、188.0707、178.0857、160.0746、132.0810	Alkaloids	a, b, c, d, e, f, g, h

87	18.72	triptoquinone A	C20H24O4	+H	329.17477	0.1	329.1744、311.1658、287.1285、283.1698、269.1526、243.1384、241.1224、229.1226、205.1225、163.0759、149.0605、69.0737	Diterpenoids	a, b, c, d, e, f, g, h
88	19.3	Tripterygiumine P	C35H41NO15	+H	716.25475	-0.2	716.2516、698.2392、688.2545、680.2285、668.2362、656.2318、594.2168、576.2054、534.1952、498.1723、326.1014、105.0351	Alkaloids	a, b, e, f, g, h
89 [a]	19.41	Triptonide*	C20H22O6	+H	359.14907	0.4	359.1485、313.1470、211.1100、197.0969、167.0850、163.0769、153.0698、115.0554、97.0663、83.0515	Diterpenoids	a, b, c, d, e, f, g, h
90	19.49	(+)-brefeldin A	C16H24O4	+H	281.17484	0.4	281.1720、263.1676、248.9912、235.1662、207.1427、193.1326、171.1099、155.0819、95.0866、85.0662、69.0730、57.0395	Others	d, f, g, h
91	19.69	wilfordinine B	C38H47NO19	+H	822.28071	-1	822.2763、804.2644、794.2806、786.2584、776.2703、734.2605、692.2530、250.1068、204.0646、194.0796、176.0693、134.0606	Alkaloids	a, b, c, d, e, f, g, h
92	20.18	Triptregelol A	C26H34O10	+H	507.22203	-0.9	507.2826、447.2020、429.1767、385.1941、345.1825、325.1641、311.1265、163.0754、137.0602、123.0410、105.0339、53.0515	Diterpenoids	a, b, c, d
93	21.01	tripterygiumine D	C33H39NO13	+H	658.24887	-0.8	658.2435、640.2343、622.2255、536.2112、518.2005、224.0924、206.0809、178.0876、160.0767、105.0356	Alkaloids	d, f, g, h
94	21.11	hypoglaside A	C36H48O11	+H	657.32633	-0.9	657.3250、625.2591、463.2471、219.1739	Triterpenoids	f, g, h

95	21.33	hypoglaunine E	C39H45NO19	+H	832.26556	-0.4	832.2602、814.2496、804.2651、786.2569、250.1075、222.0761、204.0645、194.0798、176.0698、148.0762、134.0607、95.0148	Alkaloids	a, b, c, d, e, f, g, h
96	21.49	Hypoglicin C	C19H20O3	+H	297.14872	0.7	297.1484、279.1383、269.1519、255.0993、239.0684、213.0877、	Others	a, b, c, d, e, f, g, h
97	21.53	Tripterygiumine R	C28H33NO7	+H	496.23331	0.7	496.2328、478.2207、374.2008、373.2001、356.1869、355.1943、297.1532、251.1645、149.0967、137.0966、124.0409、105.0356、78.0371、59.0574	Alkaloids	a, b, c, d, e, f, g, h
98	22.05	Tripterifordin	C20H30O3	+H	319.22658	-0.6	319.2258、301.2147、273.2079、259.1685、179.1070、155.0853、151.1123	Diterpenoids	a, b, c, d, e, f, g, h
99	22.33	Aurantiamide	C25H26N2O3	+H	403.20303	3.5	403.2067、385.1913、373.0913、252.1017、224.1079、152.1079、117.0716、105.0353、91.0567、77.0421、53.0454	Phenolic compounds	d, e, f, g
100	22.48	Triptoquinone G	C20H26O5	+H	347.18521	-0.3	347.1860、329.1744、311.1650、301.1794、287.1654、273.1475、259.1707、233.1171、219.1023、187.0771、161.0601、97.0691	Diterpenoids	a, b, c, d, e, f, g, h
101	22.73	Triptregeline C	C30H35NO10	+H	570.23304	-0.6	570.2325、552.2212、448.1986、430.1873、388.1794、124.0397、106.0324、105.0352、77.0426	Alkaloids	a, b, c, d, e, f, g, h
102	22.88	Triptregeline A	C34H39NO13	+H	670.24844	-1.5	670.2492、652.2398、634.2380、610.2306、574.2002、568.2110、548.2042、488.1927、403.1297、209.0969、137.0599、124.0395、105.0341、77.0400	Alkaloids	a, b, c, d, e, f, g, h

103	23.06	triptersinine U	C38H43NO19	+H	818.24942	-1	818.2476、800.2361、776.2397、758.2264、740.2196、716.2183、706.2344、698.2056、664.2231、646.2115、241.0856、180.0653、124.0397、95.0138	Alkaloids	a, b, c, d, e, f, g, h
104	23.11	celangulatin F	C32H42O15	+H	667.26123	2.4	667.2321、649.2584、637.1992、607.2505、501.1918、203.0750、147.0468、	Triterpenoids	c
105	23.32	evonimine	C36H43NO17	+H	762.25973	-0.9	762.2558、744.2461、734.2627、720.2471、716.2549、702.2357、660.2251、642.2150、206.0800、178.0855、160.0751、132.0810	Alkaloids	a, b, c, d, e, f, g, h
106	23.59	Triptonoterpenol	C21H30O4	+H	347.22142	-0.8	347.2212、329.2105、245.1529、231.1372、191.1061、177.0907、163.0754、69.0735	Diterpenoids	a, b, c, d, e, f, g, h
107	23.59	Tripterygiumine S	C39H43NO19	+H	830.2494	-1	830.2472、812.2354、802.2522、784.2416、770.2477、760.2428、752.2190、700.2216、658.2127、134.0600、95.0143	Alkaloids	a, b, c, d, e, f, h
108	23.69	wilfortrine	C41H47NO20	+H	874.27569	-0.8	874.2735、856.2627、846.2787、828.2680、762.2577、744.2486、250.1070、236.0914、176.0692、95.0142	Alkaloids	a, b, c, d, e, f, g, h
109	24.14	1-Desacetylwilfordine	C41H47NO18	+H	842.28577	-1	842.2811、824.2701、814.2860、796.2791、720.2483、222.0770、204.0650、194.0802、176.0702、148.0769、134.0616、105.0354	Alkaloids	a, b, c, d, e, g, h
110	24.2	Triptregeline F	C32H37NO11	+H	612.24338	-0.9	612.2416、594.2324、570.2354、552.2236、430.1873、124.0399、106.0293、105.0344、77.0412	Alkaloids	a, b, c, d, e, f, g, h

111	24.2	euonine	C38H47NO18	+H	806.28564	-1.2	806.2825、788.2718、778.2890、746.2611、704.2514、686.2413、224.0910、206.0797、178.0857、160.0749、132.0809	Alkaloids	a, b, c, d, e, f, g, h
112	24.82	regelinol	C31H48O5	+H	501.35695	-1	501.3568、483.3452、465.3353、453.3364、447.3255、423.3238、287.1994、235.1682、221.1530、201.1632、199.1474、187.1478、171.1163、157.1008、147.1171、119.0860	Triterpeno ids	a, b, c, d, e, f, g, h
113	24.93	wilforsinine A	C34H39NO12	+H	654.25408	-0.7	654.2518、612.2448、594.2335、552.2234、534.2123、490.2039、472.1954、411.1772、270.0737、147.0813、143.0864、124.0406、106.0299、105.0347、77.0418	Others	a, b, c, d, e, f, g, h
114	24.96	Triptoquinone B	C20H26O4	+H	331.19031	-0.2	331.1895、313.1793、283.1691、271.1648、255.1392、243.1374、235.1128、229.1220、215.1067、185.0959、175.1101、163.1093、155.0859、147.0447、95.0875	Diterpenoi ds	a, b, c, d, e, f, g, h
115	25.07	triptobenzene K	C20H22O5	+H	343.15392	-0.2	343.1541、325.1415、297.1487、283.0981、245.1156、231.1018、195.1062、181.0868、163.0750、149.0609、67.0593	Diterpenoi ds	a, b, c, d, e, f, g, h
116	25.18	tripterygiumine E	C39H45NO18	+H	816.26877	-2.7	816.2641、798.2553、756.2479、704.2534、644.2322、206.0805、178.0862、160.0765、132.0821	Alkaloids	a, b, c, d, e, f, g, h
117	25.2	wilfordoside A	C27H38O10	+H	523.25387	0.2	523.2511、505.2425、487.2364、375.1995、341.2168、299.1420、251.1636、193.1274、191.1435、175.1117、147.1170、131.0494	Diterpenoi ds	a, b, c, d, e, f, g, h

118	25.31	Regelone A	C28H38O5	+H	455.27876	-1	455.2548、437.2658、423.2543、411.2587、405.2410、395.2573、381.2434、353.2467、317.2267、301.2129、287.2031、239.1836、223.1616、207.1758、157.1026、69.0372	Triterpenoids	a, b, c, f, g, h
119	25.61	9′-Hydroxy-2-nicotinoylwilforine	C42H48N2O19	+H	885.29581	3.8	885.2951、867.2838、857.3000、839.2886、797.2805、250.1083、204.0659、194.0807、176.0700、105.0347	Alkaloids	a, b, c, d, e, f, g, h
120 [a]	25.65	wilfordine*	$C_{43}H_{49}NO_{19}$	+H	884.29711	-0.1	884.2955、866.2835、856.3008、848.2774、838.2901、824.2784、814.2948、806.2677、796.2806、778.2702、744.2520、734.2655、702.2413、676.2419、250.1072、236.0920、204.0653、176.0698、148.0762、134.0607、105.0342	Alkaloids	a, b, c, d, e, f, g, h
121	26.02	neotriptophenolide	C21H26O4	+H	343.19012	-0.8	343.1889、325.1803、301.1430、297.1841、283.1335、267.1042、233.1519、179.1065、163.0754、151.0751、85.0324、67.0588	Diterpenoids	a, b, c, d, e, f, g, h
122	26.05	Tripterinin	C20H30O4	+H	335.22136	-1	335.2216、317.2101、299.1998、275.2011、183.1169、171.1169、157.1011、151.0750、59.0538	Diterpenoids	a, b, c, d, e, f, g, h
123	26.17	9'-O-Acetyl-7-deacetoxy-7-oxowilfortrine	C41H45NO20	+H	872.25968	-1.3	872.2526、844.2585、760.2382、742.2289、718.2279、700.2179、278.1030、222.0760、194.0820	Alkaloids	a, b, c, d, e, h
124	26.2	Aurantiamide acetate	C27H28N2O4	+H	445.21197	-0.5	445.2128、385.1925、252.1028、224.1068、194.1176、177.0923、152.1083、134.0977、116.0642、105.0350、91.0573、77.0423	Phenolic compounds	e, f, g

125	26.22	wilfornine D	C43H49NO21	+H	916.28639	-0.6	916.2829、888.2887、804.2670、786.2568、762.2585、744.2465、316.0816、213.0915、185.0914、95.0151	Alkaloids	a, b, c, d, e, h
126	26.35	Tripterygiumine M	C39H43NO16	+H	782.26474	-0.9	782.2604、764.2556、722.2415、590.2358、206.0810、178.0869、160.0764、132.0825	Alkaloids	c, e
127 [a]	26.62	triptophenolide*	C20H24O3	+H	313.17953	-0.9	313.1790、296.1704、271.1319、267.1738、253.1227、215.1430、201.1282、165.0700、149.0942、133.0649、97.0300、81.0727	Diterpenoids	a, b, c, d, e, f, g, h
128	27.2	Triptonolide	C20H22O4	+H	327.15903	-0.2	327.1210、309.1825、299.1991、285.1483、281.1527、267.1373、257.1522、219.1373、193.1009、161.0602、137.0603、81.0727	Diterpenoids	a, b, c, d, e, f, g, h
129	27.21	19-Hydroxy-18(4→3)abeo-abieta-3,8,11, 13-tetraen-18-oic acid	C20H24O2	+H	297.18461	-1	297.1847、281.0515、269.1917、255.1382、239.1074、227.1073、203.1070、189.0923、153.0699、83.0550	Diterpenoids	a, b, c, d, e, f, g, h
130	27.22	tripterygiumine K	C39H45NO16	+H	784.28071	-0.5	784.2733、766.2680、724.2503、662.2323、644.2327、602.2128、224.0935、206.0812、178.0871、160.0765、105.0355	Alkaloids	a, b, c, e
131	27.26	triptophenolide methyl ether	C21H26O3	+H	327.19485	-1.9	327.1607、309.1825、299.1992、285.1484、281.1528、267.1373、257.1530、253.1555、219.1374、161.0600、81.0725	Diterpenoids	a, b, c, d, e, f, g, h
132	27.26	Triptonoditerpenic acid	C21H28O4	+H	345.20567	-1.1	345.2055、327.1951、309.1855、299.1997、285.1488、271.1348、257.1545、233.1537、191.1066、179.1066、163.0751、151.0762、113.0606、97.0666	Diterpenoids	a, b, c, d, e, f, g, h

133	27.62	Wilfornine A	C45H51NO20	+H	926.30417	-3.8	926.3027、898.3080、880.2992、804.2665、786.2556、762.2565、744.2456、326.1013、105.0350	Alkaloids	a, b, c, e, h
134	27.9	triptoquinone H	C20H26O3	+H	315.19518	-0.9	315.1938、285.1493、273.1492、259.1654、243.1368、231.1373、219.1373、217.1218、189.0913、177.0909、163.0750、149.0961、97.0667	Diterpenoids	a, b, c, d, e, f, g, h
135	27.99	Triptregeline D	C37H39NO13	+H	706.24716	-3.2	706.2460、688.2387、664.2382、646.2291、594.2334、584.2126、552.2221、534.2133、472.1970、435.1699、199.1122、124.0403、105.0348、95.0147、77.0414	Alkaloids	a, b, c
136 [a]	28.16	wilforine*	C43H49NO18	+H	868.30149	-0.9	868.3016、850.2905、826.2936、808.2810、790.2710、766.2740、746.2649、728.2552、704.2548、686.2428、206.0802、178.0857、160.0745、132.0811、105.0343	Alkaloids	a, b, c, d, e, f, g, h
137	28.2	wilfornine	C42H48N2O18	+H	869.30092	3.9	869.2977、851.2864、809.2786、791.2698、749.2605、686.2419、206.0816、188.0729、178.0874、160.0769、132.0827、106.0396	Alkaloids	a, b, c, d, e, f, g, h
138	28.26	ejap 4	C32H40O13	+H	633.25375	-0.7	633.2515、615.2568、207.0976、189.1288、161.0931、137.0560、87.0495、79.0574、53.0448	Triterpenoids	b, c, e
139	28.4	Tripterygiumine N	C44H45NO19	+H	892.26495	-1	892.2615、864.2688、770.2236、752.2168、728.2202、298.1080、134.0607、95.0154	Alkaloids	b, c, e
140	28.45	Tripterygiumine O	C46H49NO20	+H	936.29	-2.2	936.2867、908.2947、814.2494、796.2435、754.2359、326.1018、298.1079、123.0420、95.0157	Alkaloids	b, c, d, e, h

141	28.74	Triptobenzene A	C20H28O3	+H	317.21082	-0.9	317.2105、299.1992、275.1639、257.1533、245.1528、231.1372、219.1399、205.1213、163.0752、147.0759、133.0645、85.0677、69.0744	Diterpenoids	a, b, c, d, e, f, g, h
142	28.75	Triptobenzene D	C20H26O2	+H	299.20032	-0.8	299.2000、281.1856、257.1534、243.1372、229.1223、215.1069、201.0913、189.0918、179.1091、165.0918、147.0918、121.0663、85.0684、69.0740	Diterpenoids	a, b, c, d, e, f, g, h
143	28.85	Triptonine B	C46H49NO22	+H	968.2783	-3.7	968.2741、940.2804、856.2593、838.2490、814.2517、796.2399、744.2455、316.0815、95.0147	Others	a, b, c, e, h
144	28.94	wilforzine	C41H47NO17	+H	826.28905	-3.2	826.2860、808.2765、766.2683、704.2534、644.2313、206.0811、178.0865	Alkaloids	a, b, c, e, h
145	28.95	tripterygiumine L	C40H46N2O17	+H	827.28771	1	827.2850、809.2749、791.2670、767.2713、749.2572、206.0815、178.0876、160.0772、145.0551	Alkaloids	d, e, f, g
146	29.14	triptogelin G1	C26H34O5	+H	427.24722	-1.6	427.2467、409.2314、237.1862、219.1781、177.0564、133.0638	Diterpenoids	a, d
147	29.15	Triptregeline I	C33H35NO9	+H	590.23687	-2.7	590.2360、449.1971、355.1897、345.1687、197.1333、141.1171、124.0403、105.0356	Alkaloids	a, b, c, d, e, f, g, h
148	29.15	Wilforsinine F	C30H38O12	+H	591.24075	-4.8	591.2403、147.1202、105.0374	Triterpenoids	a, b, c, d, e, f, g, h
149	29.57	Hyponine E	C45H48N2O19	+H	921.28962	-3	921.2830、907.2724、861.2663、780.2465、206.0817、188.0718、178.0883、95.0165	Others	a, b, c, d, e, f, g, h
150	29.84	wilforol A	C29H38O5	+H	467.27834	-1.8	467.2771、449.2681、421.2720、315.2331、297.1494、219.1011、203.1811、153.0563、121.1035	Triterpenoids	a, b, c, d, e, f, g, h

151	29.96	triptohypol A	C30H40O6	+H	497.28895	-1.6	497.2863、479.2765、249.1869、231.1024、179.1084、167.1081	Triterpenoids	a, b, c, d, e, f, g, h
152	30.03	Triptonine A	C48H51NO21	+H	978.29818	-4.6	978.2970、950.3027、932.2943、866.2866、856.2603、838.2511、814.2527、796.2415、778.2370、744.2472、105.0351	Others	b, c, d, h
153	30.11	triptofordin F-4	C35H40O12	+H	653.25649	-4.2	653.2671、635.2450、593.2344、575.2197、533.2099、471.1954、293.1186、247.1312、231.0993、161.0975、131.0903、105.0363、59.0565	Triterpenoids	a, b, c, e, h
154	30.12	Wilforol E	C21H30O3	+H	331.22647	-0.9	331.2250、313.2159、299.1974、289.1806、271.1697、257.1539、245.1543、231.1378、191.0972、177.0910、163.0758、137.0601、59.0542	Diterpenoids	a, b, c, d, e, f, g, h
155	30.22	Wilforsinine H	C35H40N2O13	+H	697.26277	3.5	697.2720、655.2449、637.2453、595.2391、514.2076、124.0393、106.0291	Others	c, e
156	30.23	Wilforsinine D	C36H41NO13	+H	696.26418	-1.3	696.2641、654.2585、594.2325、576.2447、532.2253、513.2077、453.1937、124.0406、105.0353、51.0324	Others	c
157	30.29	ceanothetric acid	C30H44O7	+H	517.31499	-1.9	517.3129、499.3055、457.2959、249.1149、201.1655、187.1499、173.1337、165.0570、133.1043	Triterpenoids	a, b, c, d, f, g, h
158	30.34	triptofordin F-3	C37H42O14	+H	711.26219	-3.6	711.2618、693.2511、633.2307、591.2198、571.2143、469.1840、263.1296、205.0858、105.0359、77.0416、53.0445	Triterpenoids	a, c, h

159	30.36	Triptregeline J	C37H39NO10	+H	658.26269	-3	658.2636、491.3102、413.2010、207.1362、135.0797、124.0408、105.0356	Alkaloids	a, b, c, d, e, f, g, h
160	30.43	Triptersinine C	C34H39NO11	+H	638.25864	-1.5	638.2548、586.2522、578.2371、536.2246、518.2021、476.2070、460.1685、233.1159、191.1065、173.0975、124.0426、105.0355、77.0468	Alkaloids	e
161	30.46	triptowilfolide	C24H32O4	+H	385.23737	0.1	385.2388、367.2233、325.1827、313.1811、295.1681、271.1313、215.1079、149.0987、139.0765	Diterpenoids	c, e
162	30.61	Regeol B	C29H44O5	+H	473.32547	-1.4	473.3303、455.3226、437.3264、427.3433、413.2721、409.3195、319.2678、303.2351、237.1768、221.1557	Triterpenoids	a, b, c, d, e, f, g, h
163	30.77	wilfornine H	C44H47NO20	+H	910.27254	-4.3	910.2690、892.2595、850.2502、832.2412、790.2327、206.0814、178.0871	Alkaloids	a, b, c, e, h
164	30.81	triptofordinine A1	C41H43NO12	+H	742.28283	-4	742.2792、700.2720、682.2612、612.2419、594.2301、552.2219、534.2129、490.2066、472.1968、189.0914、173.0958、161.0967、131.0500、124.0403、105.0351、77.421	Others	a, b, c, e, f, h
165	30.86	Triptobenzene B	C20H30O2	+H	303.23172	-0.4	303.2325、285.2213、267.2098、257.2274、247.1710、233.1552、229.1596、215.1813、201.1659、165.1296、151.1125、133.1037、69.0755、57.0780	Diterpenoids	a, b, c, d, e, f, g, h
166	30.96	triregelolide A	C29H40O5	+H	469.29386	-2.1	469.2919、407.2601、235.1697、205.0871、167.1088、151.0399	Triterpenoids	a, b, c, e, f, g, h

167	31.06	Hyponine D	C47H50O18N2	+H	931.30876	-4.7	931.3062、913.2955、871.2860、853.2758、790.2655、206.0797、188.0702、160.0756、105.0353	Others	a, b, c, d, e, g, h
168	31.07	9'-O-benzoyl-lariciresinol	C27H28O7	+H	465.18898	-3.9	465.1899、447.2603、419.2583、265.1199、253.1230、235.1699、217.0876、203.1785、149.1338、135.1226、95.0867	Diterpenoids	a, e, f, g, h
169	31.34	Regelidine	C35H37NO8	+H	600.25706	-3.6	600.2545、355.1879、124.0396、105.0357、80.0531、57.0754	Others	a, b, c, d, e, f, g, h
170	31.45	Triptohairic acid	C21H28O3	+H	329.21083	-0.9	329.2111、311.2005、287.1633、283.2056、269.1531、251.1430、241.1581、229.1591、217.1602、175.1118、161.0971、147.0842、97.0668	Diterpenoids	a, b, c, d, e, f, g, h
171	31.47	wilfordinine I	C48H51NO19	+H	946.30852	-4.5	946.3074、918.3140、886.2912、824.2706、806.2631、764.2526、704.2313、250.1085、204.0657、176.0712、105.0350	Alkaloids	a, b, c, d
172 [a]	31.57	Demethylzeylasteral*	C29H36O6	+H	481.25863	0.3	481.2568、463.2488、249.1857、245.0811、233.0842、215.0714、167.1067、155.1080、117.0737	Triterpenoids	a, b, c, d, e, f, g, h
173	31.73	wilfornine C	C50H53NO20	+H	988.31892	-4.5	988.3169、960.3226、866.2798、824.2730、806.2616、746.2601、326.1007、308.0921、105.0349	Alkaloids	a, b, c, d, h
174	31.78	Regeol A	C28H40O4	+H	441.29863	-3	441.2983、423.2892、271.1698、223.1145、203.1069、189.0914、137.0506、121.1020、69.0742	Triterpenoids	a, b, c, d, e, f, g, h
175	31.78	3-Oxo-abieta-8,11,13-triene	C20H28O	+H	285.22109	-0.7	285.2202、257.2257、243.1750、225.1640、201.1637、187.1490、145.1016、141.0711、131.0863、117.0715、99.0827、85.0677、67.0594	Diterpenoids	a, b, c, d, e, f, g, h

176	31.98	tripterregeline A	C22H30O4	+H	359.22136	-0.9	359.2237、327.1955、309.1872、299.2012、285.1493、267.1359、257.1540、233.1527、191.1077、179.1073、177.0909、163.0757、97.0667、67.0593	Diterpenoids	a, b, c, e
177	32.2	tripterygiumine F	C46H49NO19	+H	920.29377	-3.7	920.2880、902.2774、860.2716、842.2594、738.2383、206.0826、178.0883、160.0785	Alkaloids	a, b, c, e, h
178	32.31	tingenin B	C28H36O4	+H	437.26782	-1.9	437.2663、419.2514、215.1066、201.0894、189.0908	Triterpenoids	a, b, c, d, e, f, g, h
179	32.42	triptofordin A	C31H36O6	+H	505.25695	-3	505.2568、487.2512、461.2659、445.2323、399.2881、255.0711、235.1715、205.1625、159.1195、133.1052、121.1039	Triterpenoids	d
180	32.43	regelindiol A	C31H50O4	+H	487.37668	-3.1	487.3751、469.3656、455.3508、451.3560、437.3403、427.3565、409.3454、341.2483、317.2469、275.1994、221.1540、215.1801、207.1739、201.1639、187.1483、147.1172、59.0529	Triterpenoids	a, b, c, d, e, f, g, h
181	32.52	Dibutyl phthalate	C16H22O4	+H	279.15882	-1	205.0856、149.0231、121.0295、57.0757	Others	a, b, c, d, e, f, g, h
182	32.66	tripterygiumine A	C50H57NO21	+H	1008.34615	-3.4	1008.3391、990.3311、868.2590、206.0824、178.0887、132.0816	Alkaloids	a, b, c, e, h
183	32.73	20-Hydroxypregn-4-en-3-one	C21H32O2	+H	317.24759	0.3	317.2451、289.2531、257.2264、161.1332、107.0885、59.0545	Diterpenoids	a, f
184	32.74	Ent-pimara-8(14),15-diene-19-ol	C20H32O	+H	289.25274	0.5	289.2499、271.2344、257.2256、217.1951、187.1486、121.0999、81.0727、59.0536、53.0409	Diterpenoids	a, b, c, d, e, f, g, h

185	32.84	orthosphenic acid	C30H48O5	+H	489.35646	-2	489.3540、471.3449、453.3348、425.3400、323.2733、309.2578、267.2096、249.1854、235.1705、187.1501、173.1344、159.1185、153.0919	Triterpenoids	a, b, c, d, e, f, g, h
186	32.88	triptotin H	C28H42O5	+H	459.31058	0.2	459.3100、441.2995、423.2886、399.2904、359.2744、293.2289、239.1804、221.1540、207.1384、123.1186、85.0688	Triterpenoids	a, b, d, e, f, g, h
187	32.93	9-O-trans-Cinnamoyl-9-debenzoylregelidine	C37H39NO8	+H	626.27289	-3.1	626.2706、485.2308、355.1921、205.1588、147.1172、131.0409、124.0393、121.0670、105.0346、80.0525	Others	a, b, c, d, e, f, g, h
188	33.13	5,8-Epidioxy-5α,8α-ergosta-6,9,22E-tien-3β-ol	C28H42O3	+H	427.32008	-1.4	427.3191、409.3094、381.3157、325.2504、287.2371、259.2424、205.1594、153.0914、137.1328、109.1031、81.0728、67.0586	Triterpenoids	a, b, c, e, f, g
189	33.26	triptohypol B	C30H40O5	+H	481.2941	-1.6	481.2925、463.2764、449.2688、263.2031、231.1024、217.0861、171.0827	Triterpenoids	a, b, c, e, f, h
190	33.45	Regeol C	C29H38O6	+H	483.27299	-2.3	483.2709、437.2687、249.1858、233.0803、217.0872	Triterpenoids	a, b, c, d, e, f, g, h
191	33.48	tingenone	C28H36O3	+H	423.28841	-2.3	403.2639、379.2276、337.2183、271.1672、215.1060、207.1801、189.0908、125.0937、83.0525、59.0528	Triterpenoids	a, b, c, e, f, g, h
192	33.58	tripterygiumine C	C48H51NO18	+H	930.31493	-3.2	930.3096、912.2994、870.2913、852.2793、748.2566、206.0813、188.0719、178.0872、160.0767、105.0362	Alkaloids	a, b, c, e, h

193	33.6	Triptocallol	C21H32O3	+H	333.24237	-0.2	333.2428、315.2336、301.2180、287.2006、283.2050、269.1908、217.1527、159.1176、149.1323、105.0711、67.0594	Diterpenoids	b, c, e, g
194	33.77	Triptocalline A	C28H42O4	+H	443.3149	-1.5	443.3125、425.3052、407.2946、397.3088、341.2486、317.2467、249.1866、235.1694、209.1541、193.1224、167.1066、153.0908、141.0914	Triterpenoids	a, b, c, d, e, f, g, h
195	34.35	wilforic acid E	C30H46O5	+H	487.34078	-2.1	487.3387、469.3297、451.3208、441.3357、423.3255、249.1853、235.1690、221.1533、153.0919	Others	a, b, c, d, e, f, g, h
196	34.37	NST6A	C32H44O5	+H	509.3229	-6.4	509.3218、491.3075、451.2847、235.1730、191.0698、155.1092	Triterpenoids	a, b, c, g, h
197	34.51	Subglutinol B	C27H38O4	+H	427.28407	-0.5	427.2856、409.2802、381.2782、287.2360、273.2257、223.1427、217.1567、205.1571、191.1051、121.0993、81.0681	Diterpenoids	a, b, c, d, e, f, g, h
198	34.65	meliavosin	C21H34O3	+H	335.25787	-0.6	335.2593、261.2174、245.2239、179.1359、131.0857、123.1208、83.0494	Diterpenoids	a, b, c, d, e, f, g, h
199 [a]	34.82	Celastrol*	C29H38O4	+H	451.28413	-0.4	451.2831、405.2780、263.2015、249.1856、215.1049、203.1787、201.0900、155.0855、133.1017	Triterpenoids	a, d, e, f, g, h
200	34.84	celastrofuran A	C29H34O5	+H	463.24766	-0.5	463.2480、281.1593、255.1534、237.1840、219.1850、191.1799、151.0758、137.0625	Triterpenoids	b, c, d, e, g
201	34.9	Triptotriterpenic acid A	C30H48O4	+H	473.36149	-2.2	473.3597、455.3502、437.3396、409.3439、383.2945、235.1688、227.1804、159.1173、119.0870、69.0739	Triterpenoids	a, b, c, d, e, f, g, h

202	34.97	triptotin F	C31H44O5	+H	497.32567	-1	497.3244、479.3154、465.3025、451.3196、437.3048、433.3072、331.2437、313.2181、263.1651、247.1694、233.1533、185.1321、171.1165、157.1016、121.1030	Triterpenoids	b, c, e, f, g, h
203	35.01	regelin	C31H48O4	+H	485.36163	-1.9	485.3598、467.3490、453.3341、425.3403、407.3287、389.3196、341.2469、321.2550、273.2209、205.1588、187.1478、145.1015、71.0532、55.0222	Triterpenoids	a, b, c, d, e, f, g, h
204	35.66	Triptonoterpene	C20H28O2	+H	301.21606	-0.5	301.2153、283.2050、273.2212、257.2262、241.1592、201.1634、165.1270、147.1171、135.1173、133.1017、67.0581	Diterpenoids	a, b, c, d, e, f, g, h
205	35.67	triptogelin E3	C31H42O7	+H	527.30143	2.1	527.3022、481.2603、455.2414、437.2340、409.2375、391.2350、301.2183、203.1815、161.1360、149.1333	Triterpenoids	a, b, d, e, h
206	35.69	Cangoronine	C30H44O5	+H	485.32564	-1.1	485.3238、467.3174、457.3290、449.3040、439.3188、421.3092、411.3246、305.2414、237.1851、221.1542、155.1022、153.0914、97.1020、83.0877、57.0388	Triterpenoids	a, b, c, d, e, f, g, h
207 [a]	36.3	wilforlide A*	C30H46O3	+H	455.35167	-0.7	455.3522、437.3416、409.3476、383.2933、367.2648、341.2495、301.2156、287.2011、247.1694、233.1542、221.1910、207.1747、179.1064、139.1103、71.0541	Triterpenoids	a, b, c, d, e, f, g, h
208	36.19	Wilforic acid B	C29H44O4	+H	457.33056	-1.5	457.3292、439.3211、411.3243、393.3144、317.2468、235.1689、153.0909、123.1171	Others	a, b, c, d, e, f, g, h

209	36.46	(4α)-Kaurane-16,18,20-triol	C20H34O3	+H	323.25779	-0.9	323.2584、305.2468、277.2164、259.2065、221.1535、173.1334、165.1290、159.1182、151.1122、137.0977、85.0685	Diterpenoids	a, b, c, d, e, f, g, h
210	36.77	Linolenic acid	C18H30O2	+H	279.23158	-1	279.2305、261.2194、209.1536、177.1648、163.1467、149.0234、137.1325、109.1022、95.0876、81.0727、67.0584	Steroids	a, b, c, d, e, f, g, h
211	36.88	triptohypol C	C29H40O4	+H	453.29929	-1.4	453.3138、435.3310、407.2936、365.2566、341.2470、271.1710、235.1687、189.0898、153.0907、123.1174、113.1078	Triterpenoids	a, b, c, e, f, g, h
212	36.91	entkauran-16β, 19-diol	C20H34O2	+H	307.26286	-1	307.2657、261.2243、251.2026、219.1736、205.1532、167.1392、137.1338、95.0878、81.0734、67.0591、55.0604	Diterpenoids	a, b, c, d, e, f, g, h
213	37.34	wilforic acid A	C29H42O4	+H	455.31456	-2.2	455.3220、437.3184、409.3109、391.3010、341.2427、319.2665、273.2551、235.1700、219.1380、205.1221、189.1638、153.0819、137.0604、121.1018	Others	a, b, c, d, e, f, g, h
214	37.95	wilforlide B	C30H44O3	+H	453.33608	-0.5	453.3347、435.3259、407.3281、355.2661、339.2322、289.2138、247.1687、233.1539、205.1575、151.1118、137.0969	Triterpenoids	a, b, c, d
215	38.25	dehydroabietane	C20H30	+H	271.24182	-0.8	271.2420、229.1969、215.1791、203.1790、189.1642、133.1012、121.1025、119.0864、69.0739、55.0599	Diterpenoids	a, b, c, d, e, f, g, h

216	38.6	monopalmitin	C19H38O4	+H	331.28406	-0.7	331.2869、313.2733、257.2493、239.2375、155.1807、149.1358、141.1643、113.1339、97.1037、85.1044、83.0887、71.0901、57.0763	Steroids	a, b, c, d, e, f, g, h
217	38.89	Linoleic acid	C18H32O2	+H	281.24719	-1.1	281.2439、263.2357、163.1474、149.1326、135.1170、123.1169、111.1177、109.1021、107.0865、97.1023、93.0719、83.0877、69.0734、55.0598	Steroids	a, b, c, d, e, f, g, h
218	39.11	pristimerin	C30H40O4	+H	465.29946	-1	465.2973、405.2787、215.1062、203.1796、201.0911、85.0309	Triterpenoids	a, b, c, d, e, f, g, h
219	39.26	Ergosterol peroxide	C28H44O3	+H	429.33628	-0.1	429.3564、411.3286、385.0890、341.2504、313.2517、305.2166、287.2015、225.1645、205.1912、193.0513、125.1347、83.0883	Lipids	d, e, f, g, h
220	40.15	Stigmasterol	C29H48O	+H	413.37731	-1.2	413.3749、395.3658、353.3210、301.1413、255.2097、177.1280、163.1503、137.1364、109.1025、95.0885、83.0890、69.0738	Lipids	a, b, c, d, e, f, g, h
221	40.17	triptotriter-penonic acid A	C30H46O4	+H	471.34559	-2.7	471.3446、453.3347、435.3247、425.3399、407.3297、249.1856、221.1544、205.1598、187.1490、173.1335、133.1025、119.0874	Triterpenoids	a, b, c, d, e, f, g, h
222	40.27	oleanoic acid 3-O-acetate	C32H50O4	+H	499.378	-0.4	499.3764、481.3688、453.3686、425.3419、413.3051、397.3083、357.2797、343.2644、329.2489、301.2189、263.2025、249.1858、235.1702、199.1498、167.1049、145.1024、83.0902、55.0634	Triterpenoids	a, b, c, d, e, f, g, h

223	40.86	Palmitic acid	C16H32O2	+H	257.24726	-1	257.2446、229.1942、201.0923、173.1525、159.1424、131.1076、117.0907、103.0755、89.0619、85.1046、75.0462、71.0895、69.0743、57.0754、55.0596	Steroids	a, b, c, d, e, f, g, h
224	41.61	Triptocallic acid B	C30H48O3	+H	457.36703	-1.3	457.3639、439.3558、421.3462、413.3789、393.3514、249.1861、235.1709、221.1903、207.1752、191.1800、113.0983、57.0398	Triterpenoids	a, b, c, d, e, f, g, h
225	41.66	(-)-16α-hydroxykauran-19-oic acid	C20H32O3	+H	321.24224	-0.6	321.2428、275.2016、247.2069、205.1251、165.1287、151.1130、137.0978、99.0831、71.0910	Diterpenoids	a, d, h
226	41.94	wilforic acid D	C32H52O5	+H	517.38719	-3	517.3506、499.3776、481.3689、453.3707、425.3502、333.2411、281.1528、267.1388、255.2161、249.1834、235.1712、213.1611、201.1657、187.1488、157.1002、85.0684	Others	a, b, c, d, f, g, h
227	42.2	hypodiol	C30H50O2	+H	443.38747	-2	443.3880、425.3776、413.3781、407.3687、369.3120、303.2685、235.2069、207.1754、203.1803、191.1809、69.0743	Triterpenoids	a, b, c, d, e, f, g, h
228	42.42	β-Daucosterol	C35H60O6	+H	577.44363	-4.6	577.4448、437.3420、279.2303、193.0480、141.1144、85.0766	Triterpenoids	a, b, c, e, f, g
229	42.7	Triptohypol E	C31H52O2	+H	457.40419	0.4	457.3564、439.3806、425.3652、411.3664、397.3528、315.2757、207.1766、141.1248、123.1119、83.0862、69.0743	Triterpenoids	a, b
230	42.73	wilforone	C30H48O2	+H	441.37211	-1.3	441.3705、423.3618、287.2374、233.1908、209.1900、205.1940、191.1798、137.1332、99.0819	Triterpenoids	a, b, c, d, e, f, g, h

231	43.29	Squalene	C30H50	+H	411.39813	-1	411.3982、355.3406、341.3156、329.3188、287.2717、273.2580、261.2594、207.2125、191.1796、123.1162、109.1024、81.0712、69.0730、55.0611	Others	a, b, d, e, g, h
232	43.54	integracin A	C37H56O8	+H	629.40289	-3	629.4296、551.3653、349.2009、307.1921、289.1802、263.2021、167.0355、163.0774、61.0347	Triterpenoids	a, c, d, e, f, h
233	44.11	Ergosterol	C28H44O	+H	397.34621	-0.7	397.3484、379.3373、299.2767、271.2451、253.1973、229.1962、189.1278、161.1344、151.1489、123.1189、109.1030、81.0740、57.0760	Lipids	a, b, c, d, e, f, g
234	44.79	Triptonoterpene methyl ether	C21H30O2	+H	315.23171	-0.5	315.2319、287.2365、283.2064、255.2103、175.1490、161.1325、147.1169、83.0874、59.0545	Diterpenoids	a, b, c, d, h
235	44.79	labd-13(E)-ene-8α, 15-diol	C20H36O2	+H	309.27809	-2.3	309.2822、263.2372、239.2020、221.2287、199.1774、195.1751、185.1575、159.1140、137.1342、123.1181、111.0837、83.0883、69.0745	Diterpenoids	a, b, c, d, e, f, g, h
236	44.99	3β-acetoxyolean-12-en-28-aldehyde	C32H50O3	+H	483.38089	-4.9	483.3801、437.3358、329.2442、285.2296、217.1651、89.0632	Triterpenoids	h
237	45.47	6-Hydroxystigmast-4-en-3-one	C29H48O2	+H	429.3723	-0.9	429.3712、411.3623、369.3135、331.3023、303.2683、275.2033、247.2443、185.1340、157.1027、111.1188、71.0901、69.0740	Triterpenoids	a, b, c, d, e, f, g, h
238	45.72	β-sitosterol	C29H50O	+H	415.39315	-0.7	415.3917、397.3842、287.2727、257.2221、163.1484、85.1047、71.0896、57.0766	Lipids	a, b, c, d, e, h

239	46.31	Zeorin	C30H52O2	+H	445.40352	-1.1	445.4009、427.3930、409.3841、371.3312、345.3128、277.2521、235.2061、207.1722、139.1085、123.1173、83.0872、69.0731	Triterpenoids	a, b, c, d, e, f, g, h
240	46.31	Friedelin	C30H50O	+H	427.39286	-1.4	427.3902、371.3306、345.3152、341.3180、277.2530、205.1945、191.1795、137.1328、123.1176、71.0522、69.0735	Triterpenoids	a, b, c, d, e, f, g, h
241	46.75	tripterfrielanon B	C31H50O3	+H	471.38246	-1.7	471.3667、439.3570、425.3639、389.3258、343.2619、273.2032、249.1841、221.1892、191.1798、171.1208、123.1180、71.0523	Triterpenoids	c, e, f
242	47.38	Soyacerebroside I	C40H75NO9	+H	714.55035	-1.6	714.5501、696.5375、552.4999、534.4859、504.4774、352.2844、280.2635、272.2582、191.1788、85.1062、57.0760	Others	a, b, c, d, e, f, g, h
243	47.93	β-Amyrin acetate	C32H52O2	+H	469.40383	-0.4	469.4045、413.3451、399.3247、331.2492、271.2453、265.2121、219.2151、209.1530、205.1944、195.1412、151.1130、143.1072、123.1194	Triterpenoids	c, d, e, f, g, h

Note: Compounds marked with “*” were confirmed by comparison with authentic standards, including retention time, accurate mass and MS/MS fragmentation behavior. Other compounds were tentatively annotated based on accurate mass, MS/MS fragments, retention behavior and literature data. a, TG; b, RTG; c, RDTG; d, MTG-1; e, MTG-2; f, MTG-3; g, MTG-4; h, MTG-5.

Table S2. Differential components between TG and RTG and between RTG and RDTG.

No.	Name	Molecular formula	Category	RTG vs. TG			RDTG vs. RTG		
				VIP value	P value	Trend	VIP value	P value	Trend
1	4-Hydroxybenzoic acid	C7H6O3	Amides	-	-	-	1.19	0.02	↓
2	vanillic acid	C8H8O4	Amides	1.57	0.00	↑	-	-	-
3	β-hydroxypropiovanillone	C10H12O4	Others	1.01	0.00	↑	-	-	-
4	syringic acid	C9H10O5	Amides	1.63	0.00	↑	-	-	-
5	Catechin	C15H14O6	Polyphenols	1.92	0.00	↓	-	-	-
6	3-hydroxy-1-(4-hydroxy-3, 5-dimethoxyphenyl)-1-propanone	C11H14O5	Others	-	-	-	1.11	0.00	↓
7	celafurine	C21H27N3O3	Alkaloids	1.13	0.00	↓	2.48	0.00	↓
8	Wilfordonol C	C13H20O3	Others	1.64	0.00	↓	-	-	-
9	vanillin	C8H8O3	Others	1.73	0.00	↑	-	-	-
10	Orcinol	C7H8O2	Others	1.84	0.00	↑	-	-	-
11	syringaldehyde	C9H10O4	Others	1.61	0.00	↑	-	-	-
12	3,4,5-Trimethoxyphenol	C9H12O4	Others	1.73	0.04	↓	-	-	-
13	3-methoxy-4-hydroxybenzenemethanol	C8H10O3	Others	1.73	0.00	↑	-	-	-
14	celabenzine	C23H29N3O2	Alkaloids	1.88	0.00	↓	2.53	0.00	↓
15	tripdiolide	C20H24O7	Diterpenoids	1.68	0.00	↓	1.64	0.01	↓
16	Isoferulaldehyde	C10H10O3	Others	2.05	0.00	↑			
17	Blumenol B	C13H22O3	Others	-	-	-	1.14	0.01	↓
18	celacarfurine	C21H25N3O4	Alkaloids	1.56	0.00	↓	-	-	-
19	wilfordoside B	C26H38O9	Diterpenoids	1.99	0.00	↓	-	-	-
20	Celallocinnine	C25H31N3O2	Alkaloids	1.13	0.00	↓	1.80	0.00	↓
21	5α-hydroxytriptonide	C20H22O7	Diterpenoids	1.24	0.00	↑	-	-	-
22	triptotin B	C20H26O6	Diterpenoids	1.25	0.00	↑	-	-	-

23	medioresinol	C21H24O7	Diterpenoids	1.87	0.00	↓	-	-	-
24	triptolide	C20H24O6	Diterpenoids	1.04	0.00	↓	-	-	-
25	(-)-olivil	C20H20O7	Diterpenoids	1.61	0.01	↑	-	-	-
26	tripfordine A	C36H45NO18	Alkaloids	-	-	-	3.20	0.00	↓
27	chiapenine ES-IV	C34H41NO17	Alkaloids	-	-	-	3.22	0.00	↓
28	Triptoquinondiol	C20H28O4	Diterpenoids	1.54	0.00	↓	-	-	-
29	triptone	C12H18O2	Others	1.17	0.00	↓	-	-	-
30	Triptersinine T	C27H35NO10	Alkaloids	1.23	0.00	↑	-	-	-
31	wilfornine E	C36H43NO18	Alkaloids	-	-	-	2.86	0.00	↓
32	Wilfordinine A	C36H45NO17	Alkaloids	-	-	-	1.31	0.00	↓
33	Tripterygiumine P	C35H41NO15	Alkaloids	-	-	-	1.32	0.00	↓
34	wilfordinine B	C38H47NO19	Alkaloids	-	-	-	2.34	0.00	↓
35	Triptregelol A	C26H34O10	Diterpenoids	1.00	0.00	↓	-	-	-
36	hypoglaunine E	C39H45NO19	Alkaloids	-	-	-	2.24	0.00	↓
37	Triptoquinone G	C20H26O5	Diterpenoids	1.16	0.00	↓	-	-	-
38	Triptregeline C	C30H35NO10	Alkaloids	1.06	0.00	↑	-	-	-
39	Triptregeline A	C34H39NO13	Alkaloids	-	-	-	1.29	0.00	↓
40	triptersinine U	C38H43NO19	Alkaloids	-	-	-	1.60	0.00	↓
41	evonimine	C36H43NO17	Alkaloids	-	-	-	1.86	0.00	↓

42	Triptonoterpenol	C21H30O4	Diterpenoids	1.21	0.00	↓	1.09	0.00	↑
43	Tripterygiumine S	C39H43NO19	Alkaloids	-	-	-	1.11	0.00	↓
44	wilfortrine	C41H47NO20	Alkaloids	-	-	-	1.66	0.00	↓
45	1-Desacetylwilfordine	C41H47NO18	Alkaloids	1.21	0.00	↑	1.39	0.00	↓
46	euonine	C38H47NO18	Alkaloids	-	-	-	1.40	0.00	↓
47	B Triptoquinone B	C20H26O4	Diterpenoids	2.08	0.00	↓	-	-	-
48	9′-Hydroxy-2-nicotinoylwilforine	C42H48N2O19	Alkaloids	1.08	0.00	↑	-	-	-
49	triptophenolide	C20H24O3	Diterpenoids	-	-	-	1.03	0.00	↑
50	Triptonolide	C20H22O4	Diterpenoids	-	-	-	1.34	0.01	↑
51	wilfornine	C42H48N2O18	Alkaloids	-	-	-	2.55	0.00	↓
52	Triptobenzene A	C20H28O3	Diterpenoids	1.93	0.00	↑	-	-	-
53	Triptobenzene D	C20H26O2	Diterpenoids	1.58	0.00	↑	-	-	-
54	Triptonine B	C46H49NO22	Others	-	-	-	1.32	0.02	↑
55	Hyponine E	C45H48N2O19	Others	-	-	-	1.14	0.00	↓
56	wilforol A	C29H38O5	Triterpenoids	1.14	0.01	↑	-	-	-
57	ceanothetric acid	C30H44O7	Triterpenoids	1.48	0.00	↓	-	-	-
58	Regeol B	C29H44O5	Triterpenoids	1.20	0.02	↑	-	-	-
59	triregelolide A	C29H40O5	Triterpenoids	1.08	0.00	↑	-	-	-
60	3-Oxo-abieta-8,11,13-triene	C20H28O	Diterpenoids	1.82	0.00	↓	-	-	-
61	tingenin B	C28H36O4	Triterpenoids	2.19	0.00	↑	-	-	-
62	Dibutyl phthalate	C16H22O4	Others	1.13	0.00	↓	1.50	0.00	↑
63	orthosphenic acid	C30H48O5	Triterpenoids	1.17	0.00	↓	-	-	-
64	tingenone	C28H36O3	Triterpenoids	-	-	-	1.00	0.04	↓

65	Triptocallol	C21H32O3	Diterpenoids	-	-	-	1.53	0.00	↑
66	wilforic acid E	C30H46O5	Others	1.19	0.00	↑	-	-	-
67	NST6A	C32H44O5	Triterpenoids	1.08	0.01	↑	-	-	-
68	Celastrol	C29H38O4	Triterpenoids	1.56	0.00	↓	-	-	-
69	A Triptotriterpenic acid A	C30H48O4	Triterpenoids	1.61	0.00	↓	-	-	-
70	triptotin F	C31H44O5	Triterpenoids	1.80	0.00	↑	1.79	0.00	↓
71	Triptonoterpene	C20H28O2	Diterpenoids	1.89	0.00	↑	-	-	-
72	wilforlide A	C30H46O3	Triterpenoids	1.02	0.00	↑	-	-	-
73	Linolenic acid	C18H30O2	Steroids	-	-	-	1.45	0.00	↓
74	wilforlide B	C30H44O3	Triterpenoids	1.56	0.00	↑	-	-	-
75	dehydroabietane	C20H30	Diterpenoids	1.16	0.02	↓	-	-	-
76	pristimerin	C30H40O4	Triterpenoids	1.18	0.00	↓	-	-	-
77	A triptotriter-penonic acid A	C30H46O4	Triterpenoids	1.08	0.00	↑	-	-	-
78	Daucosterol	C35H60O6	Triterpenoids	-	-	-	1.03	0.00	↑
79	Squalene	C30H50	Others	1.14	0.01	↓			
80	Triptonoterpene methyl ether	C21H30O2	Diterpenoids	-	-	-	1.71	0.00	↑
81	6-Hydroxystigmast-4-en-3-one	C29H48O2	Triterpenoids	1.17	0.00	↓	1.27	0.00	↑
82	Zeorin	C30H52O2	Triterpenoids	-	-	-	1.26	0.00	↑
83	Friedelin	C30H50O	Triterpenoids	-	-	-	1.16	0.00	↑
84	I Soyacerebroside I	C40H75NO9	Others	1.05	0.00	↓	-	-	-

Note: "-" indicates that the component is not a differential component in the comparison group. For RTG versus TG, compared with TG, " ↑ " indicates increased response intensity in RTG and " ↓ " indicates decreased response intensity in RTG. For RDTG versus RTG, compared with RTG, " ↑ " indicates increased response intensity in RDTG and " ↓ " indicates decreased response intensity in RDTG.

Table S3. Annotated serum-detectable prototype constituents of TG-related preparations.

NO.	T_R/min	Compounds	Molecular formula	Mode	Measured mass (m/z)	ppm	Fragment ions	Category	Source
1	4.69	wilfordic acid	$C_{11}H_{13}NO_4$	+H	224.09106	-3	224.0896、208.0382、150.0536、134.0247、118.0659	Others	a, b, c, d
2	5.76	tripterygiumine H	$C_{28}H_{37}NO_{13}$	+H	596.23438	1	596.2352、554.2345、536.2161、518.2036、224.0931、206.0824、188.0692、160.0805、132.0809	Alkaloids	a, b, c, d
3	5.92	tripdiolide*	$C_{20}H_{24}O_7$	+H	377.15939	-0.2	377.1595、341.1376、297.1137、267.1374、213.0914117.0724、85.0660	Diterpenoids	a, b, c, d
4	7.81	tripterycoside C	$C_{26}H_{32}O_{10}$	+H	505.20719	0.7	505.2047、311.1618、283.1735、191.1030、165.0942	Diterpenoids	a, b, c
5	8.1	Tripterygiumine T	$C_{32}H_{39}NO_{16}$	+H	694.23571	2.2	694.2325、620.1693、602.1919、564.2072、236.0922、208.0868、176.0713	Alkaloids	a, b, c, d
6	8.25	tripterygiumine J	$C_{34}H_{43}NO_{16}$	+H	722.26552	0.1	722.2564、668.2324、234.0816、224.0925、208.1016、105.0322	Alkaloids	a, b, c, d
7	8.32	triptolide*	$C_{20}H_{24}O_6$	+H	361.16436	-0.6	361.1666、319.1226、277.0723、263.0576、251.0553、211.0243、197.0110、167.0362、153.0203、116.9789、84.9583	Diterpenoids	a, b, c
8	8.5	macroregeline C	$C_{34}H_{41}NO_{16}$	+H	720.24774	-2.9	720.2441、702.2307、684.2483、646.2086、590.1897、234.0724、224.0917、206.0795	Alkaloids	a, b, c, d

9	9.06	Triptoquinondiol	C20H28O4	+H	333.20637	1	333.2041、315.1966、297.1822、285.1865、231.1148、215.1114、175.1105、165.0649、155.0898、147.1137、123.0839、105.0734、95.0865	Diterpenoids	a, b, c, d
10	9.09	Tripfordine A*	C36H45NO18	+H	780.26969	-1.6	780.2703、762.2616、752.2752、744.2464、734.2511、702.2435、692.2505、250.1150、236.0955、222.0656、204.0659、194.0785、176.0733	Alkaloids	a, b, c, d
11	9.28	neotriptophenolide	C21H26O4	+H	343.19034	-0.1	343.1891、325.1801、283.1290、177.1335、163.1103、153.0701、111.0823、95.0824、81.0735、67.0564、59.0518	Diterpenoids	a, b, c, d
12	9.42	16-hydroxytriptobenzene H	C21H28O5	+H	361.20111	0.5	361.2014、343.1949、315.1953、297.1836、255.1758、251.1799、153.0908、117.0754、101.0657、83.0552	Diterpenoids	a, b, c, d
13	9.58	wilfordoside A	C27H38O10	+H	523.25438	1.2	523.2590、347.2202、329.1921、311.1993、301.2149、287.1634、179.1120、131.0606	Diterpenoids	a, d
14	9.68	Tripterygiumine Q	C33H39NO16	+H	706.23474	0.8	706.2352、688.2195、646.2222、594.2191、576.2161、534.2076、528.2002、316.0878	Alkaloids	a, b, c, d
15	9.82	macroregeline D	C32H39NO15	+H	678.23979	0.8	678.2383、660.1802、206.0802、178.0874	Alkaloids	a, b

16	9.97	Triptocallol	C21H32O3	+H	333.24177	-1.9	333.2427、315.2340、297.2244、215.1537、175.1500、169.0957、161.1330、157.0917、147.1198、91.0522、69.0732	Diterpenoids	a, b, c, d
17	9.99	triptobenzene K	C20H22O5	+H	343.15402	0	343.1574、313.1506、297.1482、233.1319、195.0805、181.0991、167.0845、153.0710、125.0636、81.0724	Diterpenoids	a, b, c
18	10.11	Tripterinin	C20H30O4	+H	335.22152	-0.5	335.2113、169.1076、157.0917、151.0754	Diterpenoids	a, b, c, d
19	10.21	Wilfordinine A	C36H45NO17	+H	764.27675	0.9	764.2781、746.2646、718.2681、686.2433、206.0816、178.0877、145.0569、132.0786	Alkaloids	a
20	10.33	Tripterygiumine P	C35H41NO15	+H	716.25569	1.1	716.2558、698.2599、594.2152、576.2129、534.1998、326.1049、219.1087、175.0742、149.0580、105.0320	Alkaloids	a, c, d
21	10.67	tripterygiumine D	C33H39NO13	+H	658.25069	1.9	658.2452、640.2292、622.2302、536.2023、206.0806、178.0851、132.0815、105.0377	Alkaloids	a, b, c, d
22	10.84	Triptonide*	C20H22O6	+H	359.14867	-0.7	359.1555、209.0122、197.0138、167.0092、151.0039、110.9779、98.9757、84.9596	Diterpenoids	a
23	10.86	Triptonolide	C20H22O4	+H	327.15909	0	327.1577、299.1338、281.1514、245.1420、233.0959、219.1137、175.0755、161.0600、133.0605、113.0666	Diterpenoids	a, b, c, d

24	11.22	Tripterifordin	C20H30O3	+H	319.22657	-0.6	319.2244、301.2144、273.1869、261.1767、183.1155、169.0986、155.0843、141.0697、57.0413	Diterpenoids	a
25	11.31	ejap 4	C32H40O13	+H	633.25559	2.2	633.2541、615.2446、515.2280、455.2026、429.1670、205.0986、189.1028	Triterpenoids	a, b, c, d
26	11.46	Triptoquinone G	C20H26O5	+H	347.18504	-0.7	347.1802、329.1832、311.1646、283.1716、253.1607、225.1240、219.0977、199.1104、183.0837、173.0946、165.0670、97.0763、69.0763	Diterpenoids	a, b
27	11.73	wilforic acid A	C29H42O4	+H	455.3155	-0.2	455.3183、437.3051、395.2837、343.2397、289.1758、275.1704、237.1581、219.1705、207.1122、193.1643、189.1280、171.1146、157.0961、135.1221、121.1011	Others	b, d
28	11.84	wilfordine*	C43H49NO19	+H	884.29275	-5	884.2972、866.2939、856.2986、848.2616、838.2907、796.2678、778.2464、734.2885、204.0617、194.0875、176.0743、105.0320	Alkaloids	a, c
29	11.87	19-Hydroxy-18(4→3)abeo-abieta-3,8,11, 13-tetraen-18-oic acid	C20H24O2	+H	297.1847	-0.7	297.1848、279.1732、251.1764、237.1249、213.1316、185.1308、181.0998、167.0844、153.0700、131.0856、117.0701、81.0725、67.0586	Diterpenoids	a, b, c, d
30	11.93	Triptoquinone B	C20H26O4	+H	331.19047	0.3	331.1854、313.1795、285.1499、229.1248、175.0796、161.0585、155.0862、117.0707、95.0869、67.0586	Diterpenoids	a, b, c, d

31	11.96	wilfornine D	C43H49NO21	+H	916.28772	0.8	916.2908、804.2740、762.2849、744.2755、617.1727、95.0190	Alkaloids	a, b
32	12.12	euonine	C38H47NO18	+H	806.28739	1	806.2891、788.2751、728.2591、206.0807、188.0683、178.0871、160.0744	Alkaloids	a, b, c, d
33	12.22	wilforzine	C41H47NO17	+H	826.29305	1.7	826.2947、748.2599、704.2731、644.2213、224.0965、206.0806、178.0848、132.0842、105.0290	Alkaloids	a, c, d
34	12.26	triptophenolide	C20H24O3	+H	313.17961	-0.7	313.1791、295.1673、271.1353、267.1777、253.1225、229.1445、181.0984、165.0700、147.0820、133.1033、81.0672	Diterpenoids	a, b, c, d
35	12.31	Triptobenzene D	C20H26O2	+H	299.20026	-1	299.1994、257.1506、241.1208、229.1199、215.1092、201.0895、187.0764、179.1064、151.0730、121.0994、117.0655、67.0576	Diterpenoids	a, b, c, d
36	12.31	triptophenolide methyl ether	C21H26O3	+H	327.19527	-0.6	327.1949、309.1852、299.1992、285.1485、267.1379、257.1529、255.0979、243.1360、229.1212、219.1363、161.0592、97.0659、81.0720、57.0382	Diterpenoids	a, b, c, d
37	12.31	Triptonoditerpenic acid	C21H28O4	+H	345.20573	-0.9	345.2038、327.1937、299.1989、257.1517、233.1528、177.0901、163.0746、151.0743、97.0658、83.0507	Diterpenoids	a, b, c, d
38	12.32	triptoquinone H	C20H26O3	+H	315.19517	-1	315.1935、273.1485、255.1386、215.1431、189.1279、171.1111、161.0949、149.0968、123.0829、97.0661	Diterpenoids	a, b, c, d

39	12.51	wilforine*	C43H49NO18	+H	868.30021	-2.3	868.3026、850.2942、826.2825、808.2685、790.2559、746.2623、728.2541、704.2727、686.2416、206.0803、178.0846、132.0972、105.0348、77.0413	Alkaloids	a, b, c, d
40	12.64	Triptonoterpene methyl ether	C21H30O2	+H	315.23224	1.2	315.2340、271.2071、239.1831、231.1709、177.1282、149.0958、97.0659、85.0666、67.0562	Diterpenoids	a, b, d
41	12.73	tirptotriterpenic acid A	C30H48O4	+H	473.36287	0.7	473.3632、455.3515、427.3510、409.3418、341.2515、301.2177、275.2025、221.1547、201.1638、187.1484、173.1320、133.1013、119.0859	Triterpenoids	a, b, c, d
42	12.86	triptoquinone A	C20H24O4	+H	329.17438	-1.1	329.1733、311.1633、283.1683、269.1164、241.1202、229.1225、217.1222、205.1203、183.0785、175.0743、165.0703、163.0741、153.0684、149.0594、113.0608、67.0585	Diterpenoids	a, b, c, d
43	13.22	Triptonoterpene	C20H28O2	+H	301.21616	-0.1	301.2165、283.1992、241.1639、169.1064、161.1357、155.0812、137.0949、133.1028、67.0613、55.0590	Diterpenoids	a, b, c, d
44	13.23	wilforol A	C29H38O5	+H	467.27917	-0.1	467.2788、231.1021、217.0860、203.1792、157.0615、121.1008	Triterpenoids	b, c, d

45	13.31	wilforlide B	C30H44O3	+H	453.33591	-0.9	453.3391、435.3266、407.3313、313.2490、287.1996、271.2087、247.1663、235.1665、231.1731、221.1581、219.1340、205.1584、139.0755、83.0860	Triterpenoids	a, b, c, d
46	13.56	triptohypol A	C30H40O6	+H	497.28934	-0.9	497.2913、479.2792、449.2254、249.1863、231.1017、179.1100、153.0923、87.1019	Triterpenoids	b, c, d
47	13.63	demethylzeylasteral*	C29H36O6	+H	481.25751	-2	481.2578、463.2463、435.2456、417.2538、249.1867、231.0652、215.0715、157.0669、135.1163	Triterpenoids	a, b, c, d
48	13.65	Triptohairic acid	C21H28O3	+H	329.21184	2.2	329.2144、283.2128、269.1565、241.1569、229.1547、217.1563、175.1106、161.0938、153.0910、147.0792、115.0567、98.9862、97.0662	Diterpenoids	a, b, d
49	13.76	β-sitosterol	C29H50O	+H	415.38993	-8.5	415.4131、397.2905、317.2860、225.1871、165.1282、141.1366、123.1114、69.0705	Lipids	e
50	13.81	tripterregeline A	C22H30O4	+H	359.22078	-2.5	359.3702、341.1815、327.1962、309.1891、299.1982、285.1491、267.1365、257.1524、233.1529、191.1103、177.0907、163.0729、97.0633、67.0558	Diterpenoids	b, d
51	14.26	orthosphenic acid	C30H48O5	+H	489.35705	-0.8	489.3567、471.3440、453.3373、425.3411、389.3156、249.1852、235.1681、187.1466、173.1309、159.1169、153.0910、99.0822、73.0623	Triterpenoids	a, b, c, d

52	14.26	triptotriter-penonic acid A	C30H46O4	+H	471.34649	-0.8	471.3460、453.3369、435.3257、425.3413、407.3306、389.3227、369.2813、243.2504、287.2006、275.2068、249.1845、221.1533、205.1584、173.1318、133.1017、119.0862、83.0849	Triterpenoids	a, b, c, d
53	14.29	Regeol C	C29H38O6	+H	483.2735	-1.3	483.2745、437.2716、249.1842、233.0800、221.1885、155.0974、121.1027	Triterpenoids	a, c, d
54	14.86	wilforic acid E	C30H46O5	+H	487.34226	0.9	487.3419、249.1866、235.1684、221.1539、185.1343、167.1067、155.1107、113.0961	Others	a, b, c, d
55	14.89	Stigmasterol	C29H48O	+H	413.37421	-8.7	413.3665、351.2905、315.2696、235.1723、193.1576、141.1274、123.1131、95.0838、81.0742、57.0731	Lipids	d
56	15.09	Celastrol*	C29H38O4	+H	451.28339	-2	451.2799、433.2677、405.2682、297.1836、283.1693、251.1419、215.1113、201.0900、189.0901、157.1008、135.1128	Triterpenoids	a, d
57	15.41	Cangoronine	C30H44O5	+H	485.32552	-1.3	485.3232、467.3193、439.3223、411.3213、317.2400、235.1687、185.1266、153.0903、97.0606	Triterpenoids	a, b, c, d
58	15.79	wilforlide A*	C30H46O3	+H	455.35156	-0.9	455.3521、437.3382、409.3485、341.2502、287.1910、273.1935、247.1684、233.1877、207.1729、151.1100、141.0943、97.1063、57.0396	Triterpenoids	a, b, c, d

59	15.83	Wilforic acid B	C29H44O4	+H	457.33086	-0.8	457.3297、439.3222、411.3268、393.3154、317.2472、235.1693、191.1819、123.1161、85.0688	Others	a, b, c, d
60	15.92	pristimerin	C30H40O4	+H	465.29924	-1.5	465.2984、419.2958、283.1711、271.1691、215.1060、169.1047、133.0975、129.0700	Triterpenoids	d
61	18.19	regelindiol A	C31H50O4	+H	487.37775	-0.9	487.3767、469.3692、437.3353、409.3410、273.1830、263.0456、221.1515、207.1333、201.1622、187.1473、145.0994、119.0843、57.0741	Triterpenoids	a, b, c, d
62	18.69	Triptocallic acid B	C30H48O3	+H	457.36725	-0.8	457.3675、439.3542、411.3622、393.3474、235.1687、221.1575、191.1806、141.0894、113.0968、57.0392	Triterpenoids	a, b, c, d
63	19.95	Ent-pimara-8(14),15-diene-19-ol	C20H32O	+H	289.2518	-2.7	289.2503、271.2392、245.2263、205.2011、187.1509、147.1167、133.0993、121.1027、81.0806、69.0714、57.0737	Triterpenoids	a, b, d

Note: Compounds marked with “*” were confirmed by comparison with available authentic standards. Other serum constituents were tentatively annotated based on accurate mass, MS/MS fragments, retention behavior and literature data. a, TG; b, RTG; c, RDTG; d, MTG.
